# Restoring glucose balance: Conditional HMGB1 knockdown mitigates hyperglycemia in a Streptozotocin induced mouse model

**DOI:** 10.1016/j.heliyon.2023.e23561

**Published:** 2023-12-12

**Authors:** Zeyu Liu, Gowtham Annarapu, Hamza O. Yazdani, Qinge Wang, Silvia Liu, Jian-Hua Luo, Yan-Ping Yu, Baoguo Ren, Matthew D. Neal, Satdarshan P. Monga, Roberto Ivan Mota Alvidrez

**Affiliations:** a Trauma and Transfusion Medicine Research Center, Department of Surgery, University of Pittsburgh School of Medicine, Pittsburgh, PA 15213, USA; b Vascular Medicine Institute, University of Pittsburgh, Pittsburgh, PA 15213, USA; c Department of Surgery, University of Pittsburgh School of Medicine, Pittsburgh, PA 15213, USA; d McGowan Institute for Regenerative Medicine, University of Pittsburgh, Pittsburgh, PA 15213, USA; e Department of Pathology, University of Pittsburgh, Pittsburgh, PA 15213, USA; f Pittsburgh Liver Research Center, University of Pittsburgh, Pittsburgh, PA 15213, USA; g Department of Medicine, University of Pittsburgh, Pittsburgh, PA 15213, USA; h Pharmaceutical Sciences, College of Pharmacy, University of New Mexico, Albuquerque, NM, 87131, USA

**Keywords:** HMGB1, Hyperglycemia, Diabetes mellitus, Inflammation, RNA sequencing

## Abstract

Diabetes mellitus (DM) poses a significant global health burden, with hyperglycemia being a primary contributor to complications and high morbidity associated with this disorder. Existing glucose management strategies have shown suboptimal effectiveness, necessitating alternative approaches. In this study, we explored the role of high mobility group box 1 (HMGB1) in hyperglycemia, a protein implicated in initiating inflammation and strongly correlated with DM onset and progression. We hypothesized that HMGB1 knockdown will mitigate hyperglycemia severity and enhance glucose tolerance. To test this hypothesis, we utilized a novel inducible HMGB1 knockout (iHMGB1 KO) mouse model exhibiting systemic HMGB1 knockdown. Hyperglycemic phenotype was induced using low dose streptozotocin (STZ) injections, followed by longitudinal glucose measurements and oral glucose tolerance tests to evaluate the effect of HMGB1 knockdown on glucose metabolism. Our findings showed a substantial reduction in glucose levels and enhanced glucose tolerance in HMGB1 knockdown mice. Additionally, we performed RNA sequencing analyses, which identified potential alternations in genes and molecular pathways within the liver and skeletal muscle tissue that may account for the in vivo phenotypic changes observed in hyperglycemic mice following HMGB1 knockdown. In conclusion, our present study delivers the first direct evidence of a causal relationship between systemic HMGB1 knockdown and hyperglycemia in vivo, an association that had remained unexamined prior to this research. This discovery positions HMGB1 knockdown as a potentially efficacious therapeutic target for addressing hyperglycemia and, by extension, the DM epidemic. Furthermore, we have revealed potential underlying mechanisms, establishing the essential groundwork for subsequent in-depth mechanistic investigations focused on further elucidating and harnessing the promising therapeutic potential of HMGB1 in DM management.

## Highlights

• The first in vivo study reveals causal association between HMGB1 and hyperglycemia.
• HMGB1 knockdown presents as a potent therapeutic target for hyperglycemia in DM.
• In-depth RNA sequencing illuminates potential HMGB1 mechanisms of action.
• Findings establish a solid foundation for subsequent mechanistic investigations.

## 1 Introduction

Diabetes mellitus (DM) constitutes a complex global disease characterized by a high prevalence rate, with its development frequently attributed to factors such as obesity, genetic predisposition, and unhealthy lifestyle choices [[1], [2], [3]]. By 2035, the global prevalence of DM is projected to almost double compared to 2013, with an estimated 600 million affected individuals worldwide [4]. The alarming increase in DM prevalence is worrisome, as individuals with this condition face heightened risks for complications, particularly cardiovascular diseases like stroke and heart failure [[5], [6], [7]]. DM is classified into two primary forms based on clinical and biochemical features: Type 1 Diabetes (T1D) and Type 2 Diabetes (T2D) [8]. Despite their distinct pathophysiology, T1D and T2D both exhibit hyperglycemia, characterized by abnormally elevated blood glucose levels [[9], [10], [11]]. Consequently, achieving normoglycemia constitutes the most efficacious therapeutic outcome for individuals with DM, aiming to prevent the onset of diabetes-related complications [[12], [13], [14]]. However, numerous studies have highlighted substantial challenges healthcare providers face in effectively managing glucose control in DM patients, which may stem from factors such as limited healthcare access, suboptimal medication adherence, and social stigma associated with DM [[15], [16], [17], [18], [19], [20]]. Therefore, it is imperative to identify novel therapeutic targets that can approach the management of blood glucose levels from an alternative perspective, ultimately enhancing the global response to the DM epidemic.

In recent years, DM has been recognized not only as an endocrine disorder but also as a chronic inflammatory condition, unveiling a new avenue for exploring potential therapeutic targets to address hyperglycemia in DM [[21], [22], [23]]. Several studies have demonstrated an elevation in circulating inflammatory factors, such as chemokines and cytokines, in individuals with DM [22,[24], [25], [26], [27]]. These observations further suggest a potential contributory role of inflammatory factors in the pathogenesis and progression of diabetes. High Mobility Group Box 1 (HMGB1) is a well-defined proinflammatory protein that expressed ubiquitously in all cell types [28]. Our prior research has found that individuals with chronic DM display increased levels of both circulating HMGB1 and its pro-inflammatory form, acetylated HMGB1 [29]. Furthermore, multiple studies have reported an upregulation in the expression of the primary cellular receptors of HMGB1, namely the Receptor for Advanced Glycation End-Products (RAGE) and Toll-Like Receptor 4 (TLR4) [[30], [31], [32], [33]]. In addition, it has also been proposed that HMGB1 may interfere with insulin signaling through the insulin receptor/AKT pathway, resulting in insulin resistance and, consequently, disruptions in glucose metabolism [34]. However, a causal relationship between HMGB1 and DM in vivo remains unclear. This is primarily due to the lethality of global embryonic HMGB1 deletion, with hypoglycemia being a significant factor contributing to mortality in mice [35]. The occurrence of hypoglycemia in the context of global embryonic HMGB1 deletion highlights the potentially critical role HMGB1 might play in glucose metabolism. This observation piqued our interest, prompting us to delve deeper into the potential of HMGB1 as a promising therapeutic target for hyperglycemia management.

With the pioneering creation of the inducible HMGB1 knockout (iHMGB1 KO) model at the Department of Surgery at the University of Pittsburgh, we have, to our knowledge, the sole in vivo knockout model of HMGB1 in mice. This breakthrough circumvents the challenges posed by the lethality associated with the embryonic global HMGB1 knockout model. Consequently, it provides us with a unique platform to elucidate and probe the previously elusive relationship between HMGB1 and DM. Our hypothesis postulates that systemic conditional knockdown of HMGB1 in hyperglycemic mice will lead to a significant reduction in glucose levels and enhanced glucose tolerance. Furthermore, we hypothesize that RNA sequencing will identify relevant altered pathways in our model, aiding in the elucidation of the mechanism of action we seek to pursue understanding the role of HMGB1 in glucose regulation in DM. Our investigations aim to investigate the molecular pathways in which HMGB1 participates in modulating glucose control in hyperglycemia associated with DM.

## 2 Materials and methods

### 2.1 Animal model

All animal experiments were approved by the ethics committee of the University of Pittsburgh Institutional Animal Care and Use Committee (IACUC) and Animal Welfare Committee. All live vertebrate studies were carried out ethically in accordance with relevant guidelines and regulations at the University of Pittsburgh under protocol 21,018,382. All animals were housed under the Division of Laboratory Animal Resources (DLAR) facilities. At the end of the dietary intervention and experimental protocol, the animals were euthanized using an isoflurane overdose. Tissues including blood, heart, aorta, liver, spleen, muscle, and kidney were then harvested. The HMGB1 Flox and iHMGB1 KO model were developed in the Department of Surgery University of Pittsburgh as previously described [36]. Briefly, the HMGB1 Flox mouse strain was generated by inserting two Lox/P elements into introns 1 and 3, flanking exon 2 that covers the transcription initiation codon. In contrast, the iHMGB1 KO mouse strain has an additional inducible Cre transgene, driven by a ubiquitously active CAG promoter and estrogen receptor fusion protein, introduced into the HMGB1 Flox mouse strain. The fusion protein excises the floxed HMGB1 gene upon binding to an estrogen analog, such as tamoxifen (TMX), which was administered to the mice by intraperitoneal (IP) injection. In contrast, the HMGB1 Flox mice, which lack the inducible Cre recombinase, remain unresponsive to TMX injections. Consequently, in these mice, the HMGB1 gene remains intact, rendering them suitable as control animals in our experimental design.

In the present study, we employed 15 male mice for each of our in vivo studies cohorts, comprising 7 iHMGB1 KO mice and 8 HMGB1 Flox mice, each aged 6 weeks. To induce the knockdown of the HMGB1 gene, all the iHMGB1 KO and HMGB1 Flox mice in the cohort were subjected to intraperitoneal (IP) injections of TMX at a concentration of 1 mg/kg over a span of 10 days. As delineated earlier, the HMGB1 Flox mice, which lack the inducible mechanism for HMGB1 knockdown, served as the control group and remained unresponsive to the TMX injections. Subsequent to a 7-day post-TMX injection period, all mice were administered a low dose of Streptozotocin (STZ) via IP for 5 consecutive days at a dosage of 25 mg/kg. This STZ regimen is known to induce hyperglycemia. Following the final STZ injection, the hyperglycemic state was monitored and allowed to evolve over a 10-week period, as illustrated in Fig. 2.

### 2.2 Oral glucose tolerance test (OGTT) and metabolic cage analysis

These studies were conducted by Dr. Michael Jurczak in the Rodent Phenotyping Core within the Center for Metabolism and Mitochondrial Medicine in the Department of Endocrinology at the University of Pittsburgh. For OGTT, an N of 7 iHMGB1 KO mice and 8 HMGB1 Flox mice underwent a 16-h fasting period prior to the study. Initial blood glucose concentrations were measured using a commercial glucometer to establish a reference point. Subsequently, iHMGB1 KO TMX STZ and HMGB1 Flox TMX STZ mice were then administered an oral glucose solution (2 g/kg of body weight), and blood glucose levels were measured at baseline, 15, 30, 60, 90, and 120 min after glucose administration using a glucometer to assess glucose tolerance. The quantification of the Area Under the Curve (AUC) was performed to accurately represent the results of the OGTT following normalization with respect to the baseline glucose difference. The method employed in this study was adapted and modified from Jurczak et al., with some alterations to better suit the specific objectives and design of our study [37].

For development of the appropriate hyperglycemic model to test our hypothesis, we needed to decide to test the implementation of a diet intervention rather than just using low STZ alone. Therefore, we decided to test a normal diet vs high fat diet implementation using metabolic cage analysis to decide on the best approach [38]. Due to the low breeding yield of iHMGB1 KO mice, we decided to test the best experimental approach using our control mice. We therefore placed an N of 6 HMGB1 Flox mice per group randomized into three distinct groups based on their dietary and treatment conditions: Normal Chow (NC) + STZ, High Fat Diet (HFD), and STZ + HFD. Each mouse was individually housed in a metabolic cage for a duration of 24 h. This setup facilitated the measurement of energy expenditure, respiratory exchange ratio (RER), and physical activity, utilizing a comprehensive laboratory animal monitoring system. The conditions within the cages were meticulously maintained at a stable temperature of 23 °C and were subjected to a 12-h light/dark cycle.

### 2.3 Plasma HMGB1 and Cystatin C quantification

An N of 8 HMGB1 Flox TMX STZ and 7 iHMGB1 KO TMX STZ mice that have hyperglycemia developed for 10 weeks were fasted for 12 h overnight. The mice were then anesthetized using isoflurane and blood was collected by cardiac puncture using a 1 mL syringe. The collected blood was drawn into citrate-coated tubes and centrifuged at 3000 g for 15 min at 4 °C to obtain plasma. Prior to ELISA analysis, the plasma samples were diluted 1:4 with 0.9 % sodium chloride solution. Cystatin C (CST3) Mouse ELISA (Invitrogen, EMCST3) and HMGB1/HMG-1 ELISA Kit (Novusbio, Size/NBP2-62767) were performed following the manufacturer's protocol to quantify Cystatin C and HMGB1 levels in the plasma samples, respectively.

### 2.4 Plasma AST/ALT quantification

Plasma samples collected from above were thawed and diluted 1:10 for measurement of Aspartate aminotransferase (AST) and alanine aminotransferase (ALT) using DRI-CHEM (Heska, Loveland, CO), in accordance with the manufacturer's kit and recommended protocols. AST/ALT ratio was also calculated.

### 2.5 Mouse/Rat metabolic discovery Assay® Array (MRDMET12)

The same plasma samples collected for ELISA analysis were also diluted 1:2 and sent to Eve Technologies for a metabolic discovery assay. Technical duplicates were performed for each assay. A range of biomarkers were analyzed, including Amylin (active), C-Peptide 2, GIP (total), GLP-1 (active), Ghrelin (active), Glucagon, Insulin, Leptin, PP, PYY, Resistin, and Secretin.

### 2.6 Histological staining

Liver samples were obtained from the same cohort of HMGB1 Flox TMX STZ and iHMGB1 KO TMX STZ mice that have hyperglycemia developed for 10 weeks post last injection. Before harvesting, the liver was perfused with phosphate-buffered saline (PBS) and 4 % paraformaldehyde (PFA) to remove blood, preserve tissue structure, and initiate tissue fixation. Subsequently, the liver specimens were then immersed and further fixed in 4 % PFA for 4 h, followed by overnight incubation in a 30 % sucrose solution for cryoprotection. The specimens were embedded in OCT solution and cryosectioned into 10 μm-thick sections. Hematoxylin and eosin (H&E) staining (abcam, ab245880), Periodic Acid Schiff (PAS) staining (abcam, ab150680) and Oil Red O (Lipid Stain) Solution (abcam, ab223796) were performed on the same tissue sections according to the manufacturer's protocol. Bright field microscope images were captured for analysis at 10X and 20X imaging focus.

### 2.7 Immunoblot

An N of 4 HMGB1 flox TMX STZ mice and 4 iHMGB1 KO TMX STZ mice tissue lysates underwent lysis through cell lysis buffer (Cell signaling, Cat #: 9803) and brief sonication (<10secs). Resultant protein lysates supernatant were acquired post-centrifugation (14,000g) and quantified via BCA protein assay (Thermofisher, Cat #: 23225). Western blot analysis employed a Mini Gel Tank (Thermofisher, Cat #: NW2000) following manufacturer guideline, with a loading of 20 μg protein per lane. Ponceau S stain was performed on each membrane to verify protein loading, ensuring data normalization. Images of the stained membranes are available in the repository and can be provided upon request. Subsequently, the membranes were blocked using a solution of 5 % BSA in TBST for 1 h. After three 10-min washes with TBST, they were incubated with primary antibody (1:1000 dilution in 5 % BSA, HMGB1, Abcam, ab18256) overnight, ensuring gentle rocking throughout. After another series of three 10-min washes with TBST, the membranes were treated with the goat-anti rabbit secondary antibody (1:10,000 dilution in 5 % BSA, Invitrogen, 31460) for an hour at room temperature, again with gentle rocking. ImageJ software was used for Western blot analysis and band quantification.

### 2.8 Polymerase Chain Reaction (PCR)

An N of 4 HMGB1 flox TMX STZ mice and 4 iHMGB1 KO TMX STZ mice tail snips, measuring approximately 0.5 cm, were obtained following TMX injection. For comparative purposes, control Flox and C57/BL6 mice were also included in the study. Genomic DNA was isolated using the TRIzol Reagent (Invitrogen, 15596026) in accordance with the manufacturer's instructions. The region of interest within the HMGB1 gene was amplified using PCR. The resultant PCR products were then separated on a 1.5 % agarose gel via electrophoresis, run at 100V for a duration of 30 min. The bands were subsequently visualized under UV light.

### 2.9 Bulk RNA sequence analysis

Transcriptome sequencing was performed on an N of 4 HMGB1 flox TMX STZ mice and 4 iHMGB1 KO TMX STZ mice 10 weeks post TMX and STZ injection. Liver and skeletal muscle were collected from these animals. RNA was extracted using TRIzol Reagent (Invitrogen, 15596026), as per the manufacturer's protocol. Per individual mouse, quality control was performed on the raw sequencing reads by tool FastQC. Based on this, reads were trimmed by their sequencing quality and adapter sequences by tool Trimmomatic. After trimming, the surviving reads were aligned to mouse reference genome mm10 by STAR aligner. Gene count per sample were then quantified by STAR --*quantMode GeneCounts* function. After pre-processing, gene count matrix was used for downstream statistical analysis. Differential expression analysis was performed to compare iHMGB1 KO TMX STZ mice with HMGB1 Flox TMX STZ mice using R package *DESeq2*. Differentially expressed genes (DEGs) were defined by FDR = 5 % and fold-change ≥ 1.5 cutoff. Further, these DEGs were applied into Ingenuity Pathway Analysis to detect enriched pathways. Significant pathways were defined by FDR = 5 %. Gene ontology (GO) and Kyoto Encyclopedia of Genes and Genomes (KEGG) pathway enrichment analysis were performed using clusterprofiler on DEGs. Graphs were plotted by https://www.bioinformatics.com.cn/en (last accessed on Feb 13, 2023), an online platform for data analysis and visualization.

### 2.10 Statistical analysis

All statistical analyses were performed using unpaired two-tailed Student's t-tests, one-way and two-way factorial ANOVA with multiple comparisons, employing Tukey's post-hoc test where appropriate for each assay. Figure legends specify the number of replicates and samples analyzed. For the Oral Glucose Tolerance Test, levels above 600 mg/dL exceeded our detection limit and were thus recorded as 600. P-values <0.05 were deemed significant. GraphPad Prism was utilized for all statistical analyses. Data are presented as mean ± standard error (SE) unless otherwise indicated. An N: 3–8 biological replicates were analyzed per group overall in our studies, and the specific number of subjects is described for each assay. Assays were replicated 3 times each. Statistical significance was set at *: <0.05, **: 0.001–0.01, ***: 0.0001–0.001, and ****: <0.0001.

## 3 Results

### 3.1 HMGB1 knockdown and hyperglycemia model characterization

In our current study, we evaluated the efficacy of TMX injection in inducing HMGB1 knockout in iHMGB1 KO mice and HMGB1 Flox mice (Fig. 1). PCR analysis demonstrated a significant downregulation of the HMGB1 gene exclusively in the iHMGB1 KO group that received TMX injection (Fig. 2A). To further verify our model, we measured circulating HMGB1 levels in plasma samples harvested from iHMGB1 KO TMX STZ and HMGB1 Flox TMX STZ mice at the study's conclusion. The results indicated a pronounced reduction of HMGB1 in the iHMGB1 KO TMX STZ mice (Fig. 2B). Moreover, Western blot analysis conducted on tissues harvested from these mice, including the heart, liver, kidney, and lung, corroborated these findings as there was a substantial reduction of HMGB1 protein expression, by more than 70 %, in all tissue types sampled (Fig. 2C–D). To evaluate the potential effects of TMX injection on glucose metabolism and blood glucose levels, we measured fasting glucose levels before and after TMX administration in HMGB1 Flox and iHMGB1 KO mice. No significant differences were detected between the two groups (Fig. 3A). We then proceeded to determine the most optimal method for inducing the hyperglycemia phenotype by employing three conditions: High Fat Diet (HFD), STZ administration alone with normal chow (NC + STZ), and a combination of HFD and STZ (HFD + STZ) in HMGB1 Flox Mice. Fasting glucose levels were monitored weekly to track hyperglycemia progression. Both NC + STZ and HFD + STZ groups exhibited successful induction of hyperglycemia, with glucose levels exceeding 350 mg/dL. Metabolic cage analysis (MCA) was conducted on the STZ, HFD, and HFD + STZ groups. By measuring carbon dioxide production (VCO2) and oxygen consumption (VO2) and calculating the Respiratory Exchange Ratio (RER), we found that the NC + STZ group utilized a greater proportion of non-lipid sources compared to the HFD and HFD + STZ groups. This observation was supported by a higher RER value (∼0.8) in the NC + STZ group as opposed to the HFD and HFD + STZ groups (∼0.7) (Supplemental fig. 1 C-E).

**Fig. 1:**
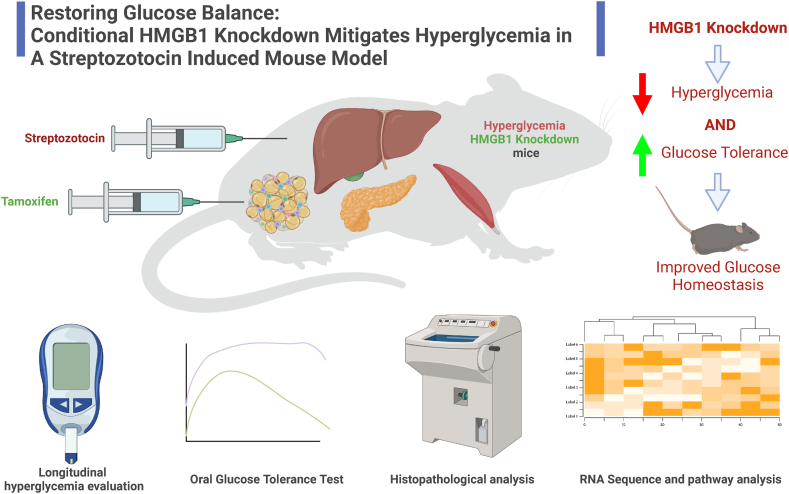
Graphical abstract has to say created with Biorender.

**Fig. 2:**
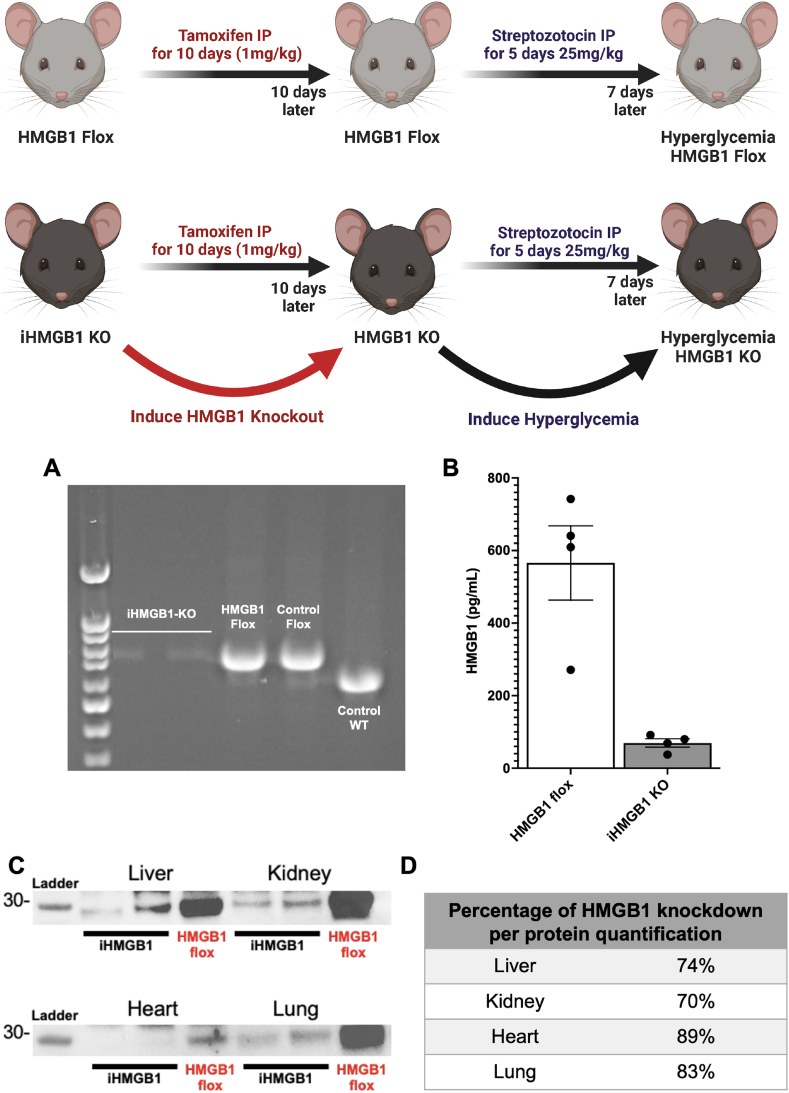
Development and efficacy of TMX-induced conditional knockdown of HMGB1 (A) Representative gel images of PCR products obtained from the tails of iHMGB1 KO TMX mice, HMGB1 Flox TMX mice, control Flox mice, and C57/BL6 mice. Our results showed that the HMGB1 gene was absent only in iHMGB1 KO mice that received TMX injection. (B) Plasma samples obtained from iHMGB1 KO TMX STZ and HMGB1 Flox TMX STZ mice were analyzed for HMGB1 concentration via ELISA. We observed a significant decrease in circulating HMGB1 in iHMGB1 KO TMX STZ mice. (C) Representative Western blot images and quantification of HMGB1 levels in different tissues (liver, kidney, heart, and lung) harvested from iHMGB1 KO TMX STZ and HMGB1 Flox TMX STZ mice showing a significant decrease in HMGB1 expression in iHMGB1 KO TMX STZ mice. (D) Immunoblot summary data of quantification of HMGB1 knockdown indicates at least 70 % of HMGB1 knockdown across all tissues following TMX injection (Full uncropped gels for protein (Fig. 2A–B) and PCR (Fig. 2C) are included as Supplemental Fig. 6 Blot Images for Fig. 2A,C). N of 4 per group were used for *t*-test analysis. Significance levels: *:<0.05, **: 0.001–0.01, ***: 0.0001–0.001, ****: <0.0001.

**Fig. 3:**
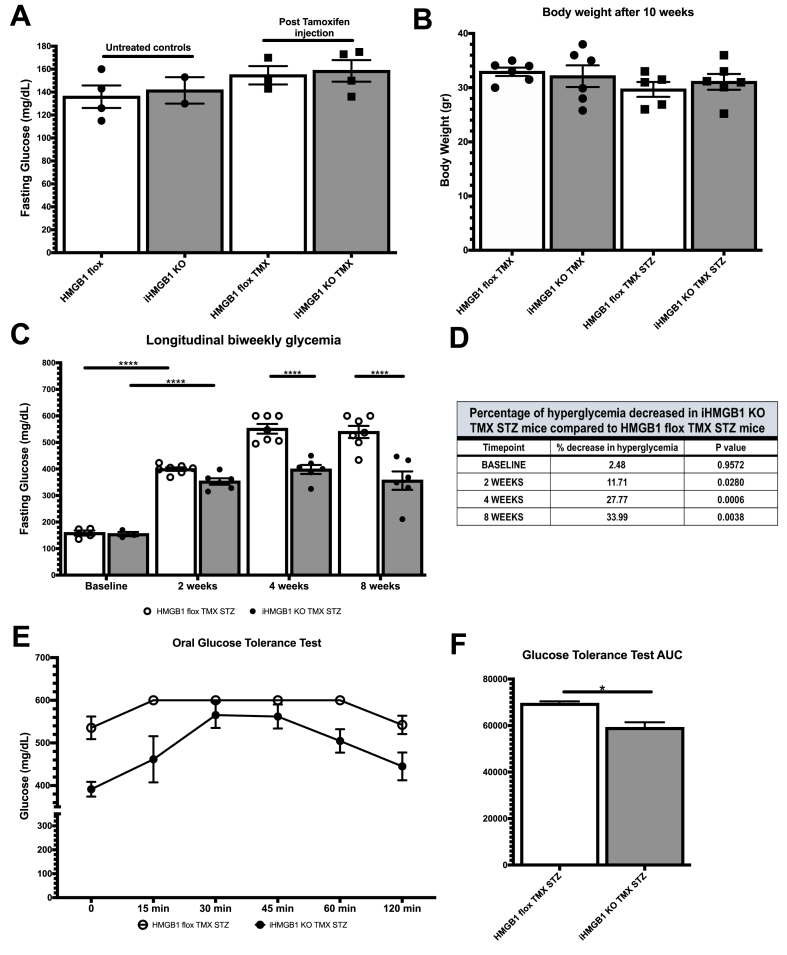
Impact of HMGB1 Knockdown on Hyperglycemia Development and Glucose Tolerance (A) Glucose levels in iHMGB1 KO TMX STZ and HMGB1 Flox TMX STZ mice show no significant difference pre- and post-TMX injection. (B) Body weight measurements of mice 10 weeks after induction of the hyperglycemia phenotype (N:7–8). (C) Longitudinal blood glucose level analysis in HMGB1 Flox TMX STZ and iHMGB1 KO TMX STZ mice following STZ injection (iHMGB1 KO TMX STZ N = 7, HMGB1 Flox TMX STZ N = 8). (D) Percent decrease in blood glucose levels compared between HMGB1 KO TMX STZ and HMGB1 Flox TMX STZ mice at various time points during hyperglycemia phenotype development. (E) Glucose tolerance test outcomes for HMGB1 KO TMX STZ and HMGB1 Flox TMX STZ mice, with 0 min denoting baseline condition and time of oral glucose administration (N = 4 per group). (F) Area under the curve (AUC) for the glucose tolerance test, normalized to baseline difference. Significance levels: *:<0.05, **: 0.001–0.01, ***: 0.0001–0.001, ****: <0.0001.

### 3.2 Knockdown of HMGB1 mitigates severe hyperglycemia during development

Upon successful model validation, we administered TMX injection followed by STZ treatment to age matched cohort of 7 iHMGB1 KO mice and 8 HMGB1 Flox mice, as illustrated in the model scheme presented in Fig. 2. To investigate the effect of HMGB1 knockdown on blood glucose levels and monitor the development of the hyperglycemia phenotype, we bi-weekly monitored fasting blood glucose levels in HMGB1 Flox and iHMGB1 KO mice after TMX and STZ administration (Fig. 3C). The results showed no difference in glucose measurements between the HMGB1 Flox TMX STZ and iHMGB1 KO TMX STZ groups at baseline or in the first two weeks after STZ injection. However, four weeks after inducing the hyperglycemia phenotype, iHMGB1 KO TMX STZ mice exhibited significantly lower glucose measurements (by 27.77 %, p = 0.0006) compared to HMGB1 Flox TMX STZ mice (Fig. 3C). As the hyperglycemia phenotype progressed, blood glucose levels of iHMGB1 KO TMX STZ mice plateaued at approximately 400 mg/dL eight weeks post-STZ treatment. In contrast, HMGB1 Flox TMX STZ mice displayed a continuous increase in blood glucose levels to around 550 mg/dL (Fig. 3C). Quantitative analysis demonstrated that iHMGB1 KO TMX STZ mice had significantly lower hyperglycemia compared to HMGB1 Flox TMX STZ mice, with a reduction of 33.99 % (p = 0.0038) eight weeks into the hyperglycemia phenotype development (Fig. 3D). To account for potential confounding factors that could affect blood glucose measurements, such as differences in food intake patterns, we assessed the body weight of both HMGB1 Flox TMX STZ and iHMGB1 KO TMX STZ mice. Our results revealed no significant difference in body weight between the two genotypes and no significant differences in body weight between mice that received only TMX and those that received both TMX and STZ (Fig. 3B). Our findings suggested that neither the knockdown of HMGB1 nor STZ injection altered food intake patterns and thereby body weight, eliminating any obvious potential confounding factors to the observed in vivo phenotype (Fig. 3B). In addition, while evaluating hormone profiling, we observed reduced levels of hormones specifically associated with digestion, including gastric inhibitory peptide (GIP) and pancreatic peptide (PP), in iHMGB1 KO TMX STZ mice (Supplemental Fig. 2). These findings indicate that HMGB1 knockdown may impact multiple aspects of metabolism and potentially influence glucose homeostasis via various pathways, including digestion.

### 3.3 Systemic knockdown of HMGB1 restored glucose tolerance by dynamic assessment of glucose tolerance

OGTT was performed in both HMGB1 Flox TMX STZ and iHMGB1 KO TMX STZ mice at ten weeks post-STZ injection. The results revealed that upon administering glucose as a stimulus, iHMGB1 KO TMX STZ mice exhibited a more gradual and controlled increase in blood glucose levels compared to HMGB1 Flox TMX STZ mice, whose blood glucose levels spiked rapidly and peaked at levels greater than 600 mg/dL, surpassing the detection limit of our standard measuring glucometer, within 15 min of administration (Fig. 3E). Furthermore, the blood glucose levels of iHMGB1 KO TMX STZ mice peaked and began to decline between 30 and 45 min post-administration, while HMGB1 Flox TMX STZ mice displayed a decreasing trend in blood glucose levels much later, at a time range between 60 and 120 min post-administration (Fig. 3E). We quantified the area under the curve (AUC) of the OGTT blood glucose curve after normalizing for baseline glucose differences. Our analysis revealed a significant reduction in AUC in iHMGB1 KO TMX STZ mice compared to HMGB1 Flox TMX STZ mice, indicating that iHMGB1 KO TMX STZ mice utilize glucose more efficiently, resulting in increased glucose tolerance (Fig. 3F).

### 3.4 HMGB1 knockdown decreases hepatic liver damage in hyperglycemic mice

In addition to evaluating glucose metabolism, we also investigated the impact of hyperglycemia reduction upon HMGB1 knockdown on the liver and kidneys, organs that play a critical role in glucose metabolism and metabolic regulation. Our assessment of the plasma samples collected from HMGB1 Flox TMX STZ and iHMGB1 KO TMX STZ mice following 10 weeks post last injection revealed that although ALT levels were comparable between the two groups, HMGB1 Flox TMX STZ mice displayed significantly elevated levels of AST, an indicator of liver damage, compared to iHMGB1 KO TMX STZ mice (Supplemental Figs. 3A and 3B). To better interpret our data, we calculated the widely used AST/ALT ratio, which showed that iHMGB1 KO TMX STZ mice had a ratio of approximately 1, whereas HMGB1 Flox TMX STZ mice exhibited a substantially higher AST/ALT ratio of around 2, suggesting that the presence of HMGB1 contributes to more severe liver damage under hyperglycemic conditions (Supplemental Fig. 3C). To further investigate the role of HMGB1 in liver damage, we examined the pathological effects of HMGB1 knockdown in the liver under a hyperglycemic state. HMGB1 Flox TMX STZ mice displayed an increase in macrovesicular lipid droplets and greater signs of mononuclear inflammatory infiltration compared to iHMGB1 KO TMX STZ mice (Supplemental Fig. 3D). Additionally, PAS and Oil Red O staining revealed that HMGB1 Flox TMX STZ mice exhibited increased glycogen storage and lipid uptake (Supplemental Figs. 3E and F). Furthermore, we evaluated the impact of HMGB1 knockdown on the kidneys, the primary organs responsible for excreting excess glucose in clinical DM patients. We measured circulating levels of Cystatin C in plasma samples obtained from HMGB1 Flox TMX STZ and iHMGB1 KO TMX STZ mice 10 weeks post hyperglycemia development. Our results indicate that iHMGB1 KO TMX STZ mice exhibited significantly elevated levels of Cystatin C compared to HMGB1 Flox TMX STZ mice (Supplemental Fig. 4).

### 3.5 Liver RNA sequencing identified significant changes in oxidative stress, lipid metabolism and autophagy related pathways

**Fig. 4:**
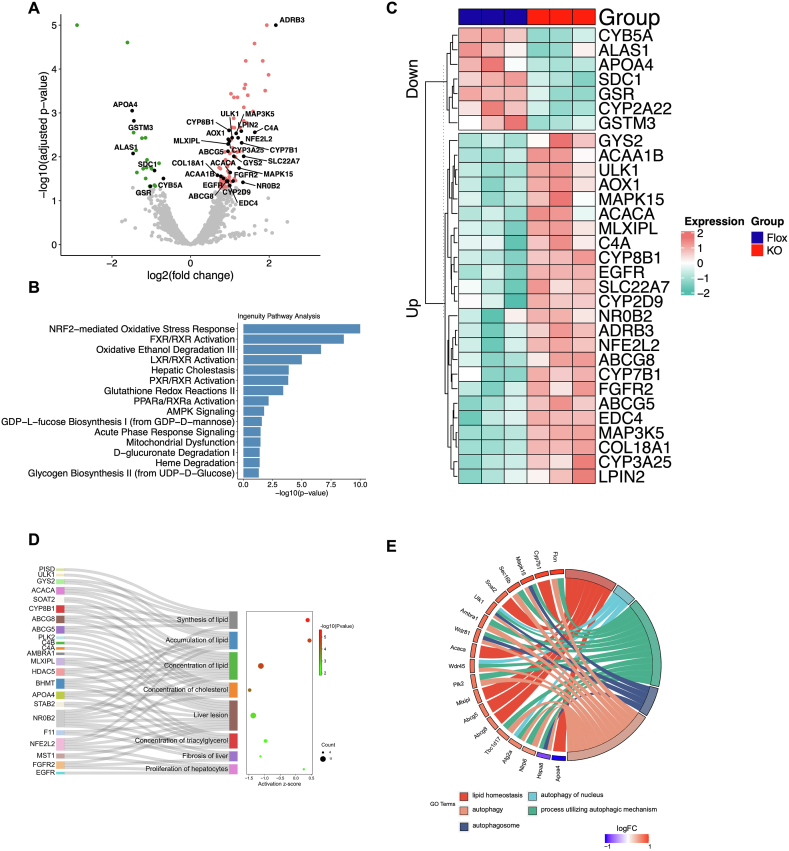
RNA sequencing analysis identifies key genes and pathways influenced by HMGB1 knockdown in the liver of hyperglycemic mice. A) Volcano plot and (C) heat map demonstrate the up- and down-regulated genes of interest upon HMGB1 knockdown, with red indicating up-regulation and blue indicating down-regulation. (B) Ingenuity Pathway Analysis (IPA) was used to identify highly enriched pathways as a result of HMGB1 knockdown using DEGs (Differentially Expressed Genes). (D) IPA analysis also revealed predictive changes in diseases/phenotypes associated with the identified DEGs, with the corresponding genes responsible shown on the left and the predicted direction of the disease/phenotype identified indicated by positive or negative Z scores shown on the right. (E) Gene ontology (GO) analysis was performed, and genes and pathways of interest are linked in a chord graph to direct further gene specific pathway studies.

Given the observed compelling in vivo phenotype and the liver's central role in metabolism, we aimed to understand the potential mechanism at play in the liver by conducting RNA sequencing on liver harvested from HMGB1 Flox TMX STZ and iHMGB1 KO TMX STZ mice 10 weeks post last injection. We performed differential expression analysis on the raw RNA sequence data and identified differentially expressed genes (DEGs), with a particular interest in genes relevant to hyperglycemia, gluconeogenesis, and diabetes. We subsequently represented these genes of interest in a volcano plot and heatmap, respectively, providing visual representations of DEGs occurred as a result of systemic HMGB1 knockdown in the liver of hyperglycemia mice (Fig. 4A, C). In addition to examining specific gene alterations, we also sought to identify and describe the key pathways impacted by HMGB1 knockdown. Therefore, we performed an Ingenuity Pathway Analysis (IPA) on the DEGs to detect enriched pathways. Notably, the NRF2-mediated oxidative stress response pathway emerged as one of the most enriched pathways, indicating a potentially more involved and altered response to HMGB1 knockdown. The FXR/LXR/RXR activation pathways, which are heavily involved in liver glucose and lipid metabolism, also shown to be highly enriched (Fig. 4B). To facilitate future mechanistic studies, we performed Gene Ontology (GO) analysis to connect genes with pathways of interest. We found that lipid homeostasis was one of the most significantly altered pathways, with ABCG5/8, MLXIPL, APOA4, and other genes closely linked to this pathway. Additionally, we identified ULK1, AYG2A, WDR45, and additional genes that contributed to multiple pathways associated with autophagy (Fig. 4E). In addition to investigating genes and pathway alterations associated with HMGB1 knockdown, we utilized IPA disease and function analysis to predict potential changes in phenotype using DEGs identified previously. Our analysis suggested with high confidence a decrease in liver lesion and liver fibrosis in iHMGB1 KO TMX STZ mice, consistent with our previous in vivo findings (Fig. 4D). In addition, we also identified a set of DEGs that are linked and responsible to the phenotypic changes observed in our study. This additional perspective offers valuable insights to inform and facilitate future mechanistic investigations.

### 3.6 RNA sequencing identified insulin signaling and glucose metabolism pathways to be altered in skeletal muscle

**Fig. 5:**
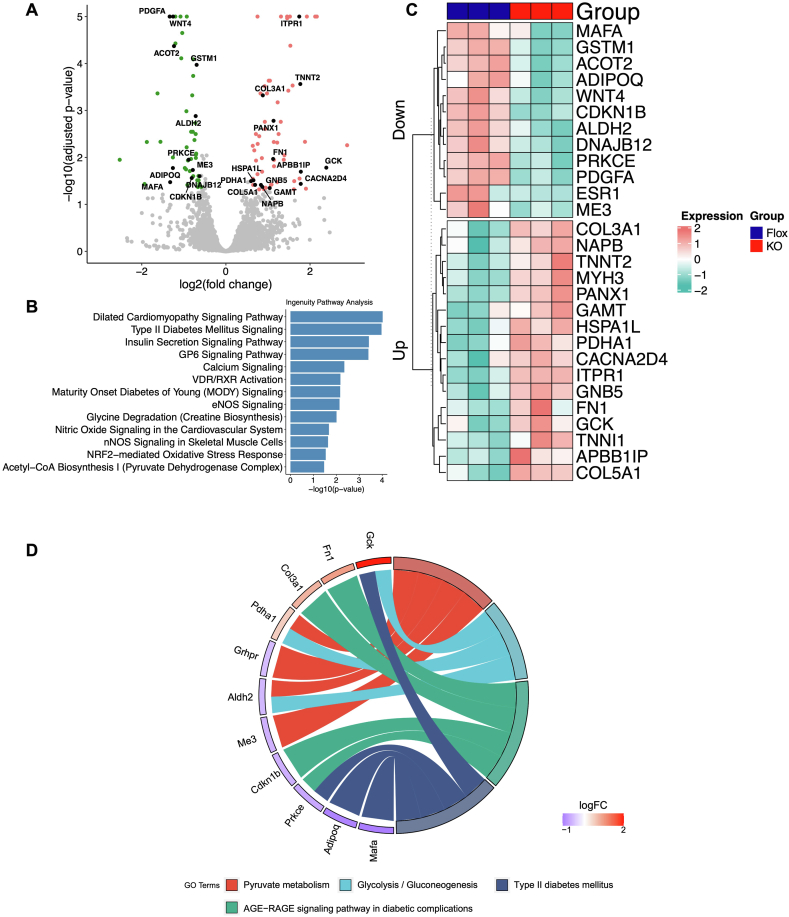
RNA sequencing analysis identifies the genes and pathways associated with DM and hyperglycemia that are impacted by HMGB1 knockdown in skeletal muscle A) A volcano plot and (C) a heat map displays the up- and down-regulated DEGs (Differentially Expressed Genes) of particular interest upon HMGB1 knockdown in hyperglycemic mice, with red indicating up-regulation and blue indicating down-regulation. (B) Ingenuity Pathway Analysis (IPA) was utilized to identify highly enriched pathways that are altered by HMGB1 knockdown in skeletal muscle. (D) Further pathway analysis using the KEGG (Kyoto Encyclopedia of Genes and Genomes) database was performed to identify additional pathways and genes of interest that are directly influenced by HMGB1 knockdown. A chord graph is used to link genes and pathways of interest, directing further studies.

To contrast the observed findings in the liver, we performed the same RNA sequencing analysis on skeletal muscle and, again, identified DEGs. We annotated key genes associated with DM and hyperglycemia and visually represented the DEGs with volcano plots and heatmaps (Fig. 5A, C). Our IPA analysis revealed unique pathways altered in skeletal muscle in iHMGB1 KO TMX STZ mice, which were distinct from those identified in the liver. Among the top altered pathways identified, most were directly associated with DM and hyperglycemia phenotypes, such as insulin secretion signaling pathways. In addition, there were also some similarly altered pathways in the skeletal muscle as those identified in the liver, such as NRF2-mediated oxidative stress response and RXR-related activation (Fig. 5B). This prompt us to compare enriched pathways identified previously with those newly identified in skeletal muscle, we found that HMGB1 appears to regulate different organs through both overlapping and distinct pathways to impact glucose metabolism (Supplemental Fig. 5). To further explore potential connections between the identified DEGs and pathways associated with skeletal muscle, we conducted Kyoto Encyclopedia of Genes and Genomes (KEGG) analysis. Our KEGG analysis revealed canonical pathways that are like those previously identified by IPA, with most closely linked to DM and hyperglycemia phenotypes. We identified key genes responsible for these changes, including GCK, PRKCE, MAFA, and Adipoq. Furthermore, we observed significant potential alternations in pyruvate metabolism and glycolysis/gluconeogenesis, with potential genes including PDHA1 and ALDH2 being responsible. Importantly, our KEGG analysis also demonstrated that the AGE-RAGE signaling pathway was significantly perturbed in skeletal muscle following HMGB1 knockdown in hyperglycemia mice. This finding is particularly noteworthy as RAGE is one of the primary canonical cellular receptors for HMGB1. We identified that FN1, COL3A1, CDKN1B, and PRKCE are among the genes associated with this signaling pathway, potentially involved in mediating HMGB1 to affect DM phenotype through RAGE (Fig. 5D).

## 4 Discussion

DM is a complex disease that involves both metabolic dysfunction and inflammation [39]. HMGB1, a proinflammatory cytokine, is elevated in DM associated hyperglycemia and inflammation [40,41]. However, most of the current research on HMGB1 and DM predominantly revolves around correlational studies [42,43]. Although some investigations demonstrated that HMGB1 inhibition has encouraging outcomes in the context of diabetes-associated pathologies [[44], [45], [46], [47]], to this day, no in vivo study has established a direct causal link between systemic HMGB1 knockdown and hyperglycemia.

This gap in the knowledge can be attributed to the lethality associated with global HMGB1 knockout mice, which results from hypoglycemia [35]. In our current study, we addressed this challenge by employing a novel TMX-induced HMGB1 KO mouse model. While the iHMGB1 KO mouse model has shown promising results in the prior study, the study only established a significant knockout of HMGB1 in brain tissue [36]. Our results revealed that TMX injection not only significantly reduces HMGB1 expression in various tissues and organs but also decreased secreted/circulating HMGB1 levels. Even though complete knockout was not observed, considerable systemic HMGB1 knockdown still warrants later studies to establish a causal relationship. We then determined the most optimal approach to induce hyperglycemic phenotype in mice. Several established methods for inducing hyperglycemia and DM phenotype exist, with HFD, low-dose STZ injection, and their combination being the most used [[48], [49], [50], [51]]. However, it remains unclear which approach is more optimal for studying glucose metabolism, as the metabolic differences between them have yet to be fully elucidated. By conducting MCA in HMGB1 Flox mice, our results indicated a decrease in RER in mice subjected to HFD and HFD + STZ, which agrees with findings in other studies that also utilized HFD [52,53]. Our interpretation of the results is also consistent with previous research that employed RER as a predictor of energy expenditure [54]. Specifically, our findings indicated that mice treated with NC + STZ exhibited a greater reliance on non-lipid sources as their primary energy source compared to mice treated with HFD or HFD + STZ. As our primary focus was to investigate the impact of HMGB1 knockdown on hyperglycemia and glucose metabolism, we opted to induce only hyperglycemia without introducing obesity and dyslipidemia by only administering low-dose STZ injections over a five-day period. We believe that this approach introduced less extraneous confounding factors to our study.

The breakthrough discovery in our current investigation was that systemic knockdown of HMGB1 in mice reduced the severity of hyperglycemia. This finding aligns with previous research suggesting that global HMGB1 KO mice succumb to hypoglycemia [35]. Notably, our longitudinal glucose measurements at baseline and the initial two weeks following STZ induction showed minimal differences between HMGB1 Flox TMX STZ and iHMGB1 KO TMX STZ mice. This observation is intriguing, as it supports the concept of HMGB1 acting as an inflammation/stress-induced cytokine. Consequently, we hypothesized that the minimal impact of HMGB1 knockdown on glucose levels in mice during periods of normoglycemia (i.e., low cellular stress) can be ascribed to the insufficient stress levels to evoke the substantial role that HMGB1 overexpression typically plays in exacerbating DM and hyperglycemia phenotypes. This speculation aligns with numerous prior studies demonstrating that HMGB1 is upregulated more prominently under conditions of high oxidative stress, a common repercussion of elevated glucose levels [[55], [56], [57]]. Our speculation was further attested as we found that blood glucose levels in iHMGB1 KO TMX STZ mice plateaued approximately four weeks after inducing the hyperglycemic phenotype, indicating that the absence of HMGB1 halted the progression of severe hyperglycemia. In contrast, glucose levels in HMGB1 Flox TMX STZ mice persisted in increasing.

Moreover, it is worth highlighting that our study found a nearly 34 % reduction in hyperglycemia in HMGB1 knockdown mice compared to hyperglycemia mice with fully expressed HMGB1. This discovery carries substantial relevance, as numerous investigations have substantiated that the attenuation of blood glucose concentrations can markedly diminish the incidence of diabetes-related complications observed in clinical contexts [58,59]. In contrast, insufficient regulation of glucose levels can exacerbate comorbidity severity [60,61]. Moreover, Metformin, the predominant pharmacological intervention to hyperglycemia in patients diagnosed with T2D, is purported to reduce glucose concentrations by approximately 30 % [62]. Consequently, a 34 % decrease in hyperglycemia indicate that targeting HMGB1 could potentially represent an equally effective, or even superior, therapeutic approach for managing hyperglycemia compared to existing interventions. Besides evaluating longitudinal glycemia measurements, which predominantly capture the effect of HMGB1 knockdown during fasting, we also performed an OGTT to assess glucose response under HMGB1 knockdown more dynamically. Glucose tolerance is also especially important to assess because clinical DM patients are known to have Impaired glucose tolerance [63] and restoring glucose tolerance has been heavily considered as a critical indicator of therapeutic efficacy [64]. Our results demonstrated that knockdown of HMGB1 restored glucose tolerance. This finding is consistent with data indicating that HMGB1 is involved in insulin resistance, potentially leading to glucose intolerance, and that HMGB1 inhibition appear to reverse this process in the retina [65,66]. Our work has also identified the potential of targeting HMGB1 to alleviate liver damage, the organ intimately involved in glucose metabolism and DM development [67,68]. In addition, our investigation extended into the kidney. We specifically looked at Cystatin C levels, a biomarker commonly used to estimate glomerular filtration rate (GFR), and assessed renal function [69]. Notably, our investigation demonstrated that HMGB1 knockdown correlated with increased Cystatin C levels, typically indicative of low GFR and high kidney damage [70]. We hypothesize that, due to the relatively brief duration of hyperglycemia development in our mouse model (10 weeks), the sampled mice are still considered to be in the early stage of diabetes, pathologically speaking. Consequently, akin to clinical research [71], only individuals with a more severe form of early diabetes exhibit elevated GFR, as observed in the HMGB1 Flox TMX STZ mice, which are likely compensating to excrete excess glucose. In contrast, since iHMGB1 KO TMX STZ mice demonstrate improved glycemic control, they do not display elevated GFR rates, consistent with a study showing that better glycemic control in patients during short-term treatment reduces GFR [72].

Liver RNA sequencing analysis revealed a marked enrichment in the NRF2-mediated oxidative stress pathway, suggesting its potential significance in mediating the phenotype observed in vivo. It is important to note that the NRF2-mediated oxidative stress pathway has not only been strongly associated with liver diseases and injury [73], but also implicated in the onset, progression, and cytokine regulation of DM [74,75]. Emerging evidence support the correlation between NRF2 and DM and posits that NRF2 may serve a protective function against the development of DM and its associated macrovascular complications [[76], [77], [78]]. In addition, HMGB1 has been demonstrated to regulate ferroptosis via NRF2, particularly in the context of high glucose levels, further highlighting the potential benefits of targeting the NRF2-mediated oxidative stress pathway in future research endeavors [79]. Besides NRF2, we also observed a significant enrichment of pathways related to FXR/LXR/PXR-RXR activation under HMGB1 knockdown in the liver. FXR and LXR have been extensively researched due to their crucial roles in glucose and lipid metabolism. Recent studies have further identified these pathways as central to the development and onset of DM, making them particularly intriguing for our future investigations [80,81]. Our current study also utilized GO analysis and established connections between genes and pathways to better direct future mechanistic studies. The results indicated that autophagy-related pathways are significantly altered in HMGB1 knockdown mice. Autophagy is a cellular process triggered by stress [82], and several studies have established its involvement in glucose metabolism [[83], [84], [85]], especially in response to high glucose [82,86]. Autophagy is particularly interesting to us also because several studies have shown that endogenous HMGB1 can influence the autophagy process. This highlights the potentially important role that autophagy may play in the observed in vivo phenotype upon HMGB1 knockdown [[87], [88], [89]].

We also investigated pathways associated with the vital role skeletal muscle plays. Interestingly, while we found some shared pathways between liver and muscle, such as the NRF2 oxidative stress response, most were unique to muscle tissue. Through IPA we identified that skeletal muscle has a more direct involvement in regulating glucose metabolism and insulin signaling, with numerous associated pathways being enriched. Our observations stand in contrast to the liver, which is primarily associated with more indirect pathways, such as oxidative stress. This finding aligns with prior research that recognizes skeletal muscle as the primary organ responsible for maintaining glucose homeostasis, contributing to over 80 % of glucose uptake [90]. Furthermore, using KEGG analysis, we discovered that the AGE-RAGE signaling pathway seems to be a key driver behind the phenotype observed in HMGB1 knockdown hyperglycemic mice. This is particularly noteworthy because RAGE is one of the primary receptors for HMGB1 [90]. Moreover, studies have reported upregulated AGE-RAGE expression in DM patients, suggesting its potential involvement in DM pathogenesis, which warrants further investigation [91]. Our study has notable limitations. First, we utilized a STZ-induced hyperglycemia mouse model, which focused exclusively on glucose metabolism due to its significant contribution to DM. While this model provided insight into the isolated role of HMGB1 in hyperglycemia, it oversimplifies the complex systemic nature of DM. Nonetheless, the findings have important clinical implications. Secondly, our study was limited to male mice, as our latest cohort revealed reduced responsiveness to STZ induction in female mice (data not shown), aligned with previous study [92]. Thirdly, our study design involved inducing HMGB1 knockdown before the onset of hyperglycemia, which limits the strength of evidence for HMGB1 as a therapeutic target in established DM. In response to the limitations of our study, our future research will build upon our current findings by investigating the effects of HMGB1 knockdown in a more complete DM phenotype that includes female mice and evaluates the therapeutic potential of targeting HMGB1 after hyperglycemia induction. Additionally, we aim to explore the potential tissue-specific effects of HMGB1 knockdown to further elucidate the exact mechanisms at play.

In conclusion, our current investigation stands as the first study to present compelling evidence that HMGB1 plays a pivotal role in regulating glucose metabolism, and that HMGB1 knockdown can significantly mitigate the severity of hyperglycemia in vivo. Our study holds particular significance, as it not only determined the potential therapeutic benefits of targeting HMGB1 to address hyperglycemia and the DM epidemic, but also represents the inaugural study to characterize promising mechanisms underlying the action of HMGB1 knockdown on hyperglycemia and DM.

## Data availability statement

The data that support the findings of this is available in the MENDELEY DATA repository: Mota Alvidrez, Roberto (2023), “Restoring Glucose Balance: Conditional HMGB1 Knockdown Mitigates Hyperglycemia in A Streptozotocin Induced Mouse Model”, Mendeley Data, V1, https://doi.org/10.17632/ryz5h94pyh.1.

## Funding

NIGMS 3R35GM119526-07S1 to RIMA; 10.13039/100008237Department of Surgery and Pittsburgh Liver Research Center P&F Award Funding for RIMA (P30 DK120531); 10.13039/100000050NHLBI R25HL145817 to RIMA; 10.13039/100006108NCATS KL2 TR001448 funding for RIMA (10.13039/100007179UNM HSC CTSC).

## Additional Information

Raw data, methods, protocols, software, hardware and other outputs – associated with the paper data is included in the MENDELEY Data Repository included in the Data Availability Statement above.

## CRediT authorship contribution statement

**Zeyu Liu:** Writing – review & editing, Writing – original draft, Software, Methodology, Investigation, Formal analysis, Data curation. **Gowtham Annarapu:** Visualization, Methodology, Investigation, Formal analysis, Data curation. **Hamza O. Yazdani:** Validation, Resources, Methodology. **Qinge Wang:** Resources. **Silvia Liu:** Validation, Software, Resources, Methodology, Formal analysis, Data curation. **Jian-Hua Luo:** Methodology, Formal analysis. **Yan-Ping Yu:** Methodology, Formal analysis, Data curation. **Baoguo Ren:** Methodology, Formal analysis. **Matthew D. Neal:** Writing – original draft, Visualization, Validation, Investigation, Funding acquisition. **Satdarshan P. Monga:** Writing – original draft, Visualization, Validation, Supervision, Resources, Methodology, Funding acquisition, Conceptualization. **Roberto Ivan Mota Alvidrez:** Writing – review & editing, Writing – original draft, Visualization, Validation, Supervision, Software, Resources, Project administration, Methodology, Investigation, Funding acquisition, Formal analysis, Data curation, Conceptualization.

## Declaration of Competing interest

The authors declare the following financial interests/personal relationships which may be considered as potential.

## Figures and Tables

**figs1:**
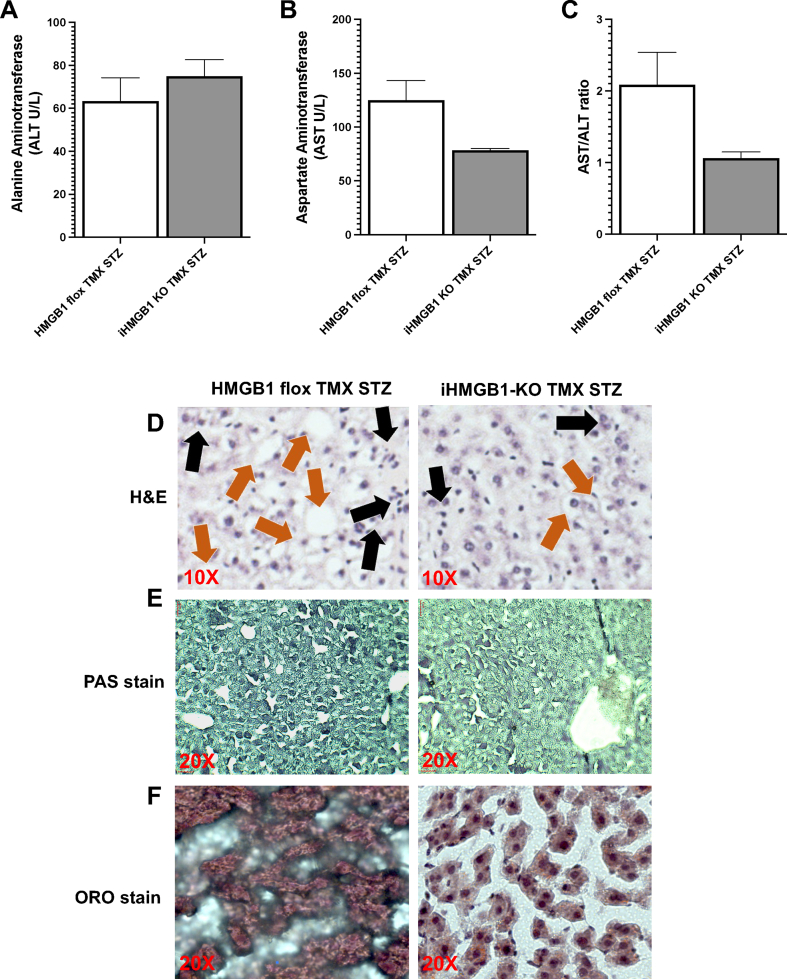
Conditional knockout of HMGB1 in diabetic mice protects from liver damage. iHMGB1-KO TMX STZ mice have decreased liver damaged evidenced by decreased AST and AST/ALT ratio (A-C), decrease inflammatory infiltrates (black arrowheads) and lipid droplets (orange arrowheads) (D), decrease glycogen storage (PAS staining) (E) and ORO staining (F) compared to HMGB1 flox TMX STZ mice.

## References

[1] Olokoba A.B., Obateru O.A., Olokoba L.B. (2012). Type 2 diabetes mellitus: a review of current trends. Oman Med. J..

[2] Chiang J.I. (2018). Associations between multimorbidity, all-cause mortality and glycaemia in people with type 2 diabetes: a systematic review. PLoS One.

[3] Kharroubi A.T., Darwish H.M. (2015). Diabetes mellitus: the epidemic of the century. World J. Diabetes.

[4] Forouhi N.G., Wareham N.J. (2014). Epidemiology of diabetes. Medicine (Abingdon).

[5] Khan M.A.B. (2020). Epidemiology of type 2 diabetes - global burden of disease and Forecasted trends. J Epidemiol Glob Health.

[6] De Rosa S. (2018). Type 2 diabetes mellitus and cardiovascular disease: genetic and Epigenetic links. Front. Endocrinol..

[7] Tomic D., Shaw J.E., Magliano D.J. (2022). The burden and risks of emerging complications of diabetes mellitus. Nat. Rev. Endocrinol..

[8] Khawandanah J. (2019). Double or hybrid diabetes: a systematic review on disease prevalence, characteristics and risk factors. Nutr. Diabetes.

[9] Wondmkun Y.T. (2020). Obesity, insulin resistance, and type 2 diabetes: essociations and therapeutic implications. Diabetes Metab Syndr Obes.

[10] Thomas D.D. (2019). Hyperinsulinemia: an early indicator of metabolic dysfunction. Journal of the Endocrine Society.

[11] Tan S.Y. (2019). Type 1 and 2 diabetes mellitus: a review on current treatment approach and gene therapy as potential intervention. Diabetes Metabol. Syndr.: Clin. Res. Rev..

[12] Nathan D.M. (2009). Medical management of hyperglycemia in type 2 diabetes: a consensus algorithm for the initiation and adjustment of therapy: a consensus statement of the American Diabetes Association and the European Association for the Study of Diabetes. Diabetes Care.

[13] Magkos F., Hjorth M.F., Astrup A. (2020). Diet and exercise in the prevention and treatment of type 2 diabetes mellitus. Nat. Rev. Endocrinol..

[14] Pasquel F.J. (2021). Management of diabetes and hyperglycaemia in the hospital. Lancet Diabetes Endocrinol..

[15] Chepulis L. (2021). Barriers to diabetes Self-management in a subset of New Zealand sdults with type 2 diabetes and poor glycaemic control. J. Diabetes Res..

[16] Aschner P. (2020). Persistent poor glycaemic control in individuals with type 2 diabetes in developing countries: 12 years of real-world evidence of the International Diabetes Management Practices Study (IDMPS). Diabetologia.

[17] Khan H., Lasker S.S., Chowdhury T.A. (2011). Exploring reasons for very poor glycaemic control in patients with Type 2 diabetes. Primary Care Diabetes.

[18] Tong W.T., Vethakkan S.R., Ng C.J. (2015). Why do some people with type 2 diabetes who are using insulin have poor glycaemic control? A qualitative study. BMJ Open.

[19] Afroz A. (2019). Glycaemic control for people with type 2 diabetes mellitus in Bangladesh - an urgent need for optimization of management plan. Sci. Rep..

[20] Edelman S.V., Polonsky W.H. (2017). Type 2 diabetes in the real world: the elusive nature of glycemic control. Diabetes Care.

[21] Calle M.C., Fernandez M.L. (2012). Inflammation and type 2 diabetes. Diabetes Metab..

[22] Tsalamandris S. (2019). The role of inflammation in diabetes: current concepts and future perspectives. Eur. Cardiol..

[23] Lontchi-Yimagou E. (2013). Diabetes mellitus and inflammation. Curr Diab Rep.

[24] Donath M.Y., Shoelson S.E. (2011). Type 2 diabetes as an inflammatory disease. Nat. Rev. Immunol..

[25] Randeria S.N. (2019). Inflammatory cytokines in type 2 diabetes mellitus as facilitators of hypercoagulation and abnormal clot formation. Cardiovasc. Diabetol..

[26] King G.L. (2008). The role of inflammatory cytokines in diabetes and its complications. J. Periodontol..

[27] Shi J. (2019). Cytokines and abnormal glucose and lipid metabolism. Front. Endocrinol..

[28] Yang H., Wang H., Andersson U. (2020). Targeting inflammation driven by HMGB1. Front. Immunol..

[29] Liu Z. (2022). Type-2 diabetic/obese patients with associated Vascular disease have increased circulating acetylated HMGB1. Faseb. J..

[30] Sabbatinelli J. (2022). Circulating levels of AGEs and soluble RAGE isoforms are associated with all-cause mortality and development of cardiovascular complications in type 2 diabetes: a retrospective cohort study. Cardiovasc. Diabetol..

[31] Dasu M.R. (2010). Increased voll-like receptor (TLR) activation and TLR tigands in recently diagnosed type 2 diabetic subjects. Diabetes Care.

[32] Ramasamy R., Yan S.F., Schmidt A.M. (2011). Receptor for AGE (RAGE): signaling mechanisms in the pathogenesis of diabetes and its complications. Ann. N. Y. Acad. Sci..

[33] Manigrasso M.B. (2014). Unlocking the biology of RAGE in diabetic microvascular complications. Trends Endocrinol Metab.

[34] Jiang Y., Steinle J.J. (2018). HMGB1 inhibits insulin signalling through TLR4 and RAGE in human retinal endothelial cells. Growth Factors.

[35] Calogero S. (1999). The lack of chromosomal protein Hmg1 does not disrupt cell growth but causes lethal hypoglycaemia in newborn mice. Nat. Genet..

[36] Aneja R.K. (2019). Lack of benefit on brain Edema, blood-brain barrier eermeability, or pognitive outcome in global inducible high mobility group box 1 knockout mice cespite tissue dparing after experimental sraumatic brain injury. J. Neurotrauma.

[37] Jurczak M.J. (2011). SGLT2 deletion improves glucose homeostasis and preserves pancreatic beta-cell function. Diabetes.

[38] Edmunds L.R. (2020). Liver-specific Prkn knockout mice are more susceptible to diet-induced hepatic steatosis and insulin resistance. Mol. Metabol..

[39] Tsalamandris S. (2019). The role of inflammation in diabetes: current concepts and future perspectives. European cardiology.

[40] Wang H., Qu H., Deng H. (2015). Plasma HMGB-1 levels in subjects with obesity and type 2 diabetes: a Cross-cectional study in China. PLoS One.

[41] Yu Y. (2015). The role of high mobility group box 1 (HMGB-1) in the diabetic retinopathy inflammation and apoptosis. Int. J. Clin. Exp. Pathol..

[42] Yan X.X. (2009). Increased serum HMGB1 level is associated with coronary artery disease in nondiabetic and type 2 diabetic patients. Atherosclerosis.

[43] Zhao D. (2013). Increased serum HMGB1 related with HbA1c in coronary artery disease with type 2 diabetes mellitus. Int. J. Cardiol..

[44] Yang L. (2020). Inhibition of HMGB1 involved in the protective of salidroside on liver injury in diabetes mice. Int. Immunopharm..

[45] Wang W.-k. (2014). Inhibition of high-mobility group box 1 improves myocardial fibrosis and dysfunction in diabetic cardiomyopathy. Int. J. Cardiol..

[46] Liu B. (2019). Inhibition of HMGB1 sromotes psseointegration under hyperglycemic condition through omprovement of BMSC dysfunction. Oxid. Med. Cell. Longev..

[47] Wang C. (2016). Inhibiting HMGB1 reduces ierebral ischemia reperfusion injury in diabetic mice. Inflammation.

[48] Premilovac D. (2017). A new method for targeted and custained induction of type 2 diabetes in sodents. Sci. Rep..

[49] Furman B.L. (2021). Streptozotocin-induced diabetic models in mice and rats. Current Protocols.

[50] Morris J.L. (2016). Development of a diet-induced murine model of diabetes featuring cardinal metabolic and pathophysiological abnormalities of type 2 diabetes. Biol Open.

[51] Skovsø S. (2014). Modeling type 2 diabetes in rats using high fat diet and streptozotocin. J Diabetes Investig.

[52] Bhandarkar N.S. (2021). Adaptation of fuel selection to acute decrease in voluntary energy expenditure is governed by dietary macronutrient composition in mice. Physiological Reports.

[53] White A.T. (2015). Knockout of STAT3 in skeletal muscle does not prevent high-fat diet-induced insulin resistance. Mol. Metabol..

[54] Rothschild J.A. (2022). Factors influencing Substrate oxidation during submaximal cycling: a sodelling analysis. Sports Med..

[55] Yu Y., Tang D., Kang R. (2015). Oxidative stress-mediated HMGB1 biology. Front. Physiol..

[56] Albert-Garay J.S., Riesgo-Escovar J.R., Salceda R. (2022). High glucose concentrations induce oxidative stress by inhibiting Nrf2 expression in rat Müller retinal cells in vitro. Sci. Rep..

[57] Buranasin P. (2018). High glucose-induced oxidative stress impairs proliferation and migration of human gingival fibroblasts. PLoS One.

[58] Laakso M. (1999). Benefits of mtrict glucose and blood sressure control in type 2 diabetes. Circulation.

[59] Gaster B., Hirsch I.B. (1998). The effects of improved glycemic control on complications in type 2 diabetes. Arch. Intern. Med..

[60] Hulme K.D. (2020). High glucose levels increase influenza-associated damage to the pulmonary epithelial-endothelial barrier. Elife.

[61] Singh A.K., Singh R. (2020). Does poor glucose control increase the severity and mortality in patients with diabetes and COVID-19?. Diabetes Metab Syndr.

[62] Hundal R.S. (2000). Mechanism by which metformin reduces glucose production in type 2 diabetes. Diabetes.

[63] Nathan D.M. (2007). Impaired fasting glucose and Impaired glucose tolerance: implications for care. Diabetes Care.

[64] Mari A. (2006). Restoration of normal glucose tolerance in severely obese patients after bilio-pancreatic diversion: role of insulin sensitivity and beta cell function. Diabetologia.

[65] Liu L., Jiang Y., Steinle J.J. (2017). Inhibition of HMGB1 protects the retina from ischemia-reperfusion, as well as reduces insulin resistance proteins. PLoS One.

[66] Guzmán-Ruiz R. (2021). The potential role of the adipokine HMGB1 in obesity and insulin resistance. Novel effects on adipose tissue biology. Mol. Cell. Endocrinol..

[67] Zhao Y. (2022). Impaired glucose tolerance is associated with enhanced postprandial pancreatic polypeptide secretion. J. Diabetes.

[68] Mohamed J. (2016). Mechanisms of Diabetes-Induced Liver Damage: the role of oxidative stress and inflammation. Sultan Qaboos Univ Med J.

[69] Mussap M. (2002). Cystatin C is a more sensitive marker than creatinine for the estimation of GFR in type 2 diabetic patients. Kidney Int..

[70] Murty M.S. (2013). Serum cystatin C as a marker of renal function in detection of early acute kidney injury. Indian J. Nephrol..

[71] Tonneijck L. (2017). Glomerular Hyperfiltration in diabetes: mechanisms, clinical significance, and treatment. J. Am. Soc. Nephrol..

[72] Meeme A., Kasozi H. (2009). Effect of glycaemic control on glomerular filtration rate in diabetes mellitus patients. Afr. Health Sci..

[73] Zhou J., Zheng Q., Chen Z. (2022). The Nrf2 pathway in liver diseases. Front. Cell Dev. Biol..

[74] Sireesh D. (2018). Association of NF-E2 related factor 2 (Nrf2) and inflammatory cytokines in recent onset type 2 diabetes mellitus. Sci. Rep..

[75] David J.A. (2017). The hrf2/neap1/ARE pathway and oxidative stress as a therapeutic target in type II diabetes mellitus. J. Diabetes Res..

[76] Uruno A. (2013). The Keap1-Nrf2 system prevents onset of diabetes mellitus. Mol. Cell Biol..

[77] Wu J. (2020). Protective role of NRF2 in macrovascular complications of diabetes. J. Cell Mol. Med..

[78] Yagishita Y. (2019). Nrf2 represses the onset of type 1 diabetes in non-obese diabetic mice. J. Endocrinol..

[79] Wu Y. (2021). HMGB1 regulates ferroptosis through Nrf2 pathway in mesangial cells in response to high glucose. Biosci. Rep..

[80] Ding L. (2014). Coordinated actions of FXR and LXR in metabolism: from pathogenesis to pharmacological targets for type 2 diabetes. Int J Endocrinol.

[81] Calkin A.C., Tontonoz P. (2012). Transcriptional integration of metabolism by the nuclear sterol-activated receptors LXR and FXR. Nat. Rev. Mol. Cell Biol..

[82] Li Q. (2018). Inhibition of autophagy promoted high glucose/ROS-mediated apoptosis in ADSCs. Stem Cell Res. Ther..

[83] Bhattacharya D. (2018). Is autophagy associated with diabetes mellitus and its complications? A review. Excli j.

[84] Bharath L.P., Rockhold J.D., Conway R. (2021). Selective autophagy in hyperglycemia-induced microvascular and macrovascular diseases. Cells.

[85] Yao J. (2014). Regulation of autophagy by high glucose in human retinal Pigment ppithelium. Cell. Physiol. Biochem..

[86] Kobayashi S. (2012). Suppression of autophagy is protective in high glucose-induced cardiomyocyte injury. Autophagy.

[87] Tang D. (2010). Endogenous HMGB1 regulates autophagy. J. Cell Biol..

[88] Frisardi V., Matrone C., Street M.E. (2021). Metabolic eyndrome and autophagy: focus on HMGB1 protein. Front. Cell Dev. Biol..

[89] Yang K. (2023). The relationship between HMGB1 and autophagy in the pathogenesis of diabetes and its complications. Front. Endocrinol..

[90] Merz K.E., Thurmond D.C. (2020). Role of skeletal muscle in insulin resistance and glucose uptake. Compr. Physiol..

[91] Villegas-Rodríguez M.E. (2016). The AGE-RAGE Axis and its relationship to sarkers of cardiovascular disease in newly diagnosed diabetic patients. PLoS One.

[92] Kim B. (2020). Sex differences in glucose metabolism of streptozotocin-induced diabetes inbred mice (C57BL/6J). Applied Biological Chemistry.

