# Supplementary figures and images for: Restoring glucose balance: Conditional HMGB1 knockdown mitigates hyperglycemia in a Streptozotocin induced mouse model
Source: Heliyon. 2023 Dec 12;10(1):e23561. doi: 10.1016/j.heliyon.2023.e23561 (PMC10770459; doi:10.1016/j.heliyon.2023.e23561)

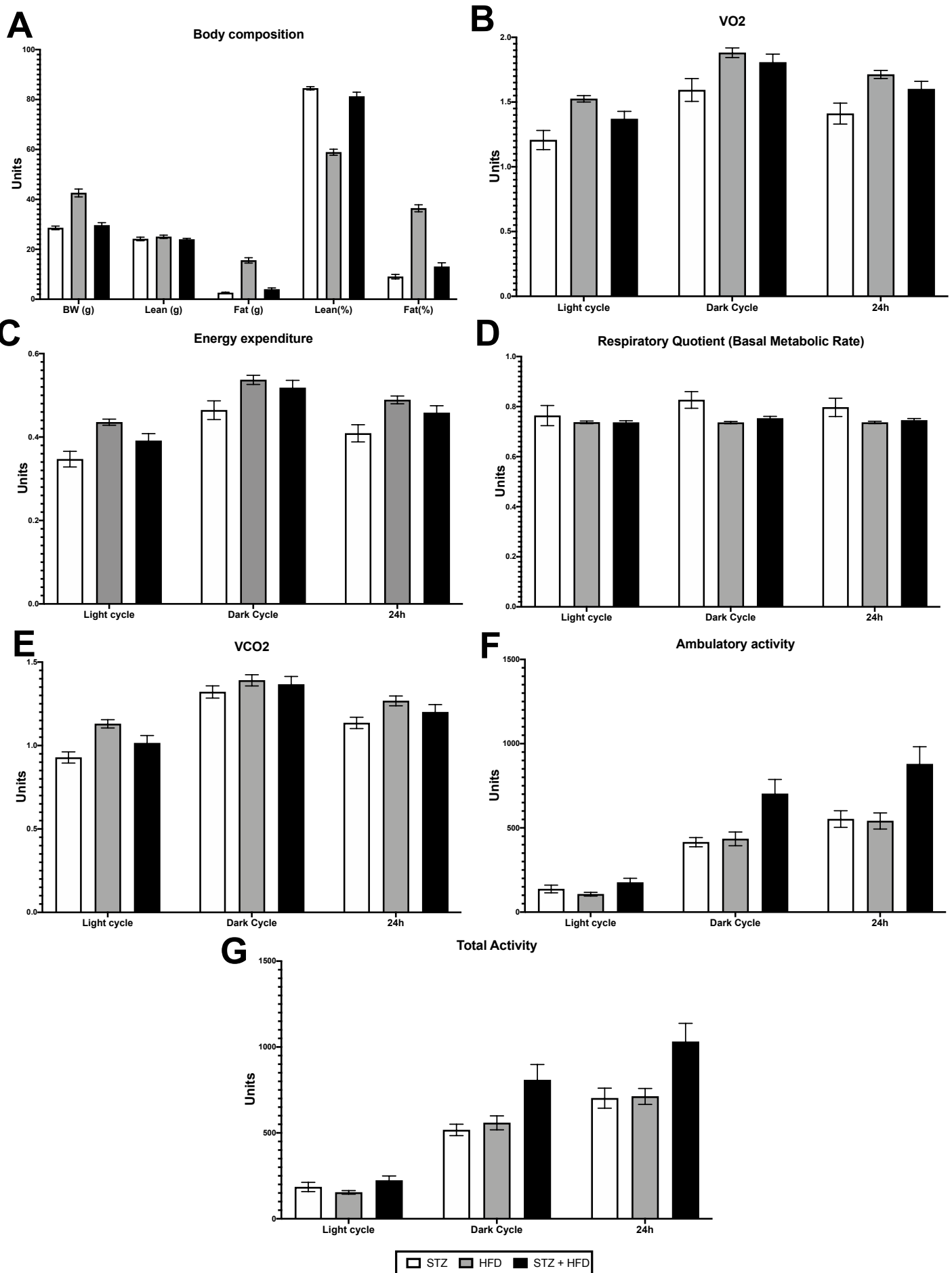

Supplement: Multimedia component 1 [file mmc1.pdf]

**A**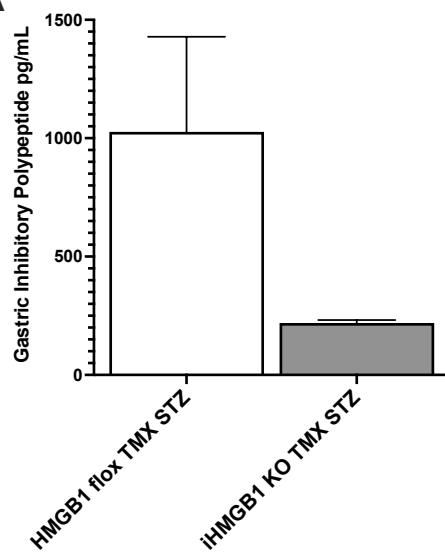**B**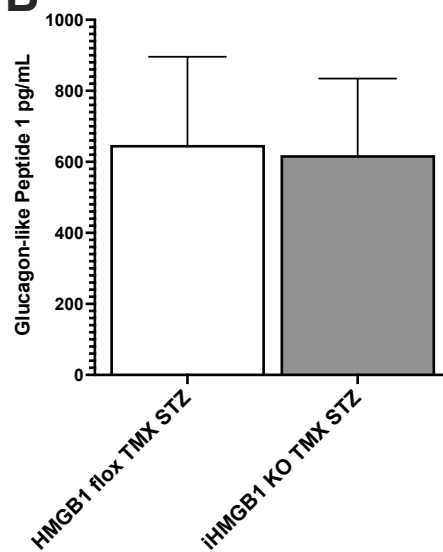**C**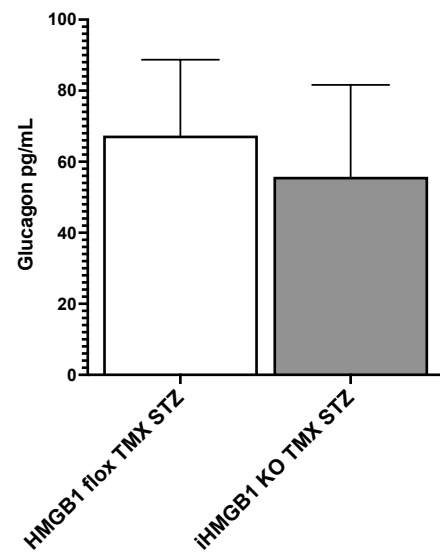**D**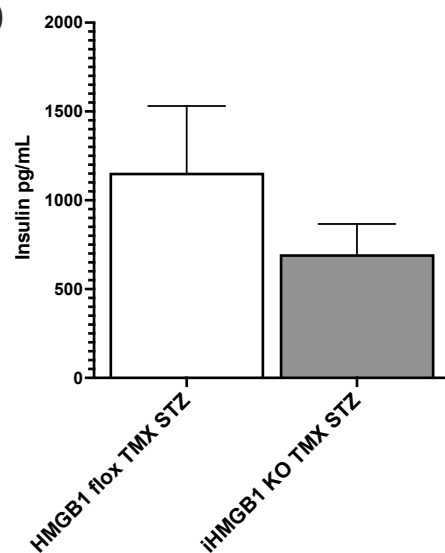**E**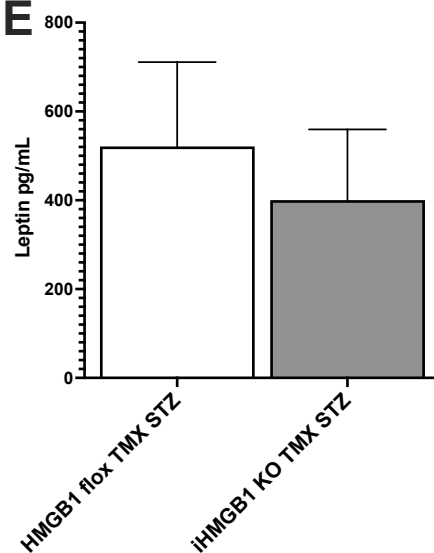**F**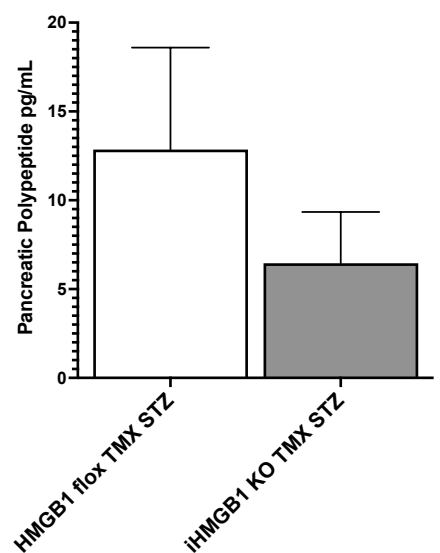**G**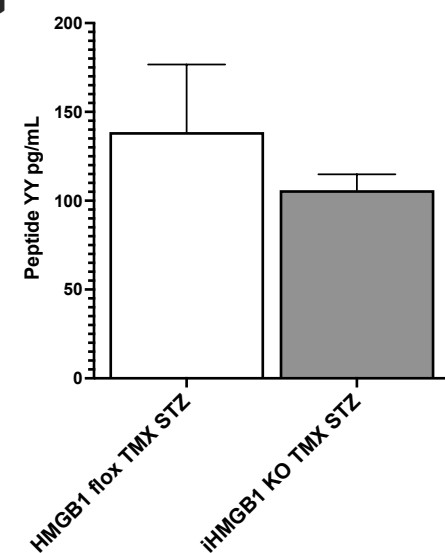**H**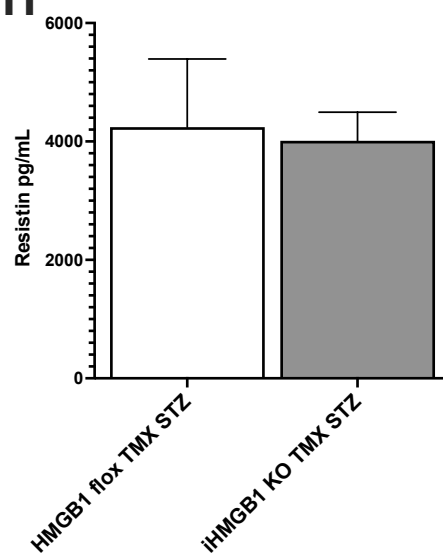**I**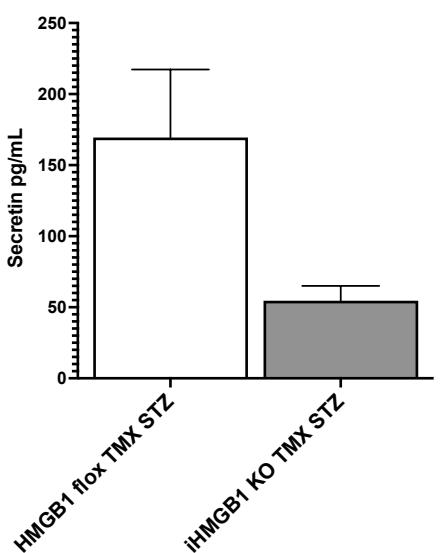

Supplement: Multimedia component 2 [file mmc2.pdf]

# Circulating Cystatin C

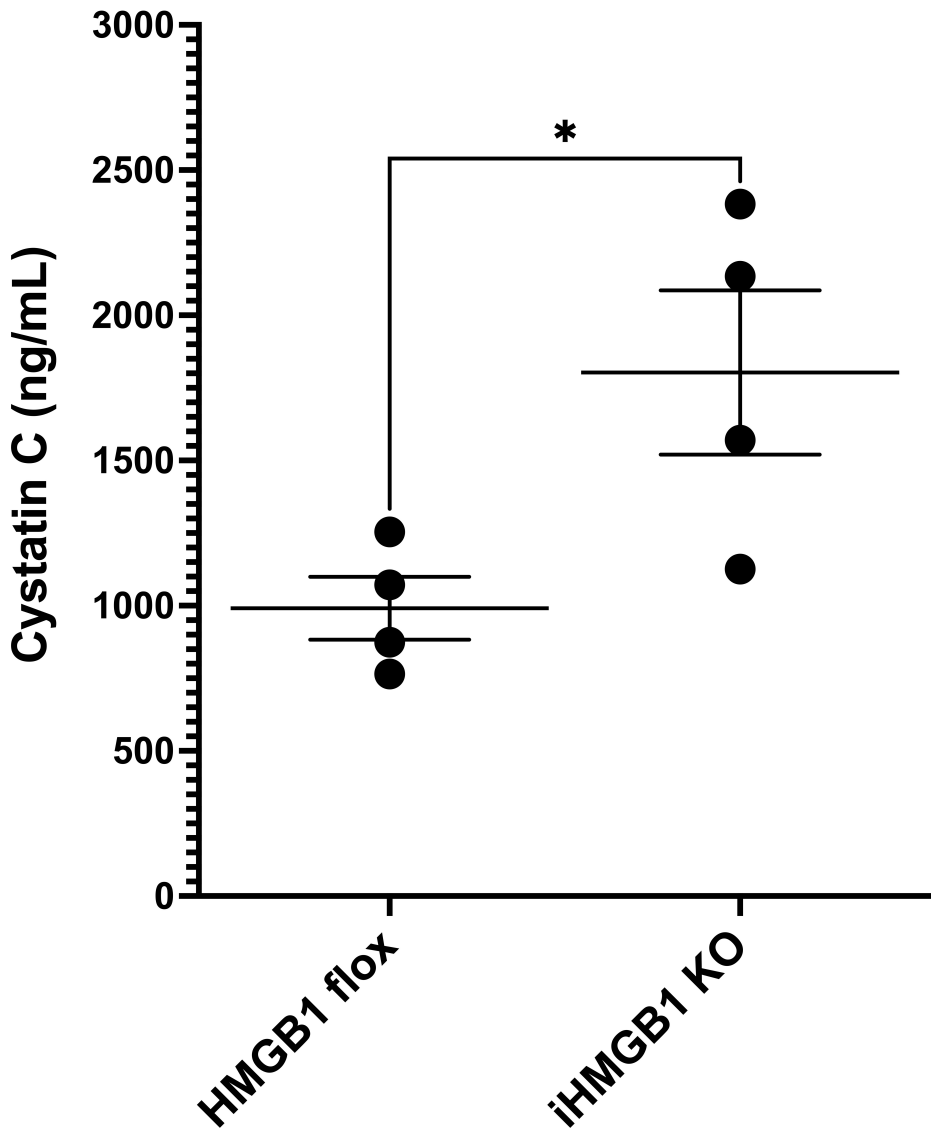

Supplement: Multimedia component 3 [file mmc3.pdf]
