# Supplementary material for: Restoring glucose balance: Conditional HMGB1 knockdown mitigates hyperglycemia in a Streptozotocin induced mouse model
Source: Heliyon. 2023 Dec 12;10(1):e23561. doi: 10.1016/j.heliyon.2023.e23561 (PMC10770459; doi:10.1016/j.heliyon.2023.e23561)
Supplement: Multimedia component 4 [file mmc4.pdf]

A

Unique Pathways for Liver

1. FXR/RXR Activation
2. Oxidative Ethanol Degradation III
3. Pregnenolone Biosynthesis
4. LXR/RXR Activation
5. Nicotine Degradation II
6. Ubiquinol-10 Biosynthesis (Eukaryotic
7. Acetone Degradation I (to Methylglyoxal
8. Nicotine Degradation III
9. Hepatic Cholestasis
10. PXR/RXR Activation
11. Glutathione Redox Reactions II
12. Histidine Degradation VI
13. Estrogen Biosynthesis
14. Œ±-tocopherol Degradation
15. Lysine Degradation II

B

Unique Pathways for Skeletal Muscle

1. Dilated Cardiomyopathy Signaling Pathway
2. Type II Diabetes Mellitus Signaling
3. Insulin Secretion Signaling Pathway
4. GP6 Signaling Pathway
5. Synaptogenesis Signaling Pathway
6. Aldosterone Signaling in Epithelial Cells
7. Phenylalanine Degradation IV (Mammalian, via Side Chain
8. G Beta Gamma Signaling
9. Calcium Signaling
10. RHOGDI Signaling
11. Hepatic Fibrosis Signaling Pathway
12. Glutamine Biosynthesis I
13. Putrescine Degradation III
14. Netrin Signaling
15. Endocannabinoid Neuronal Synapse Pathway

C

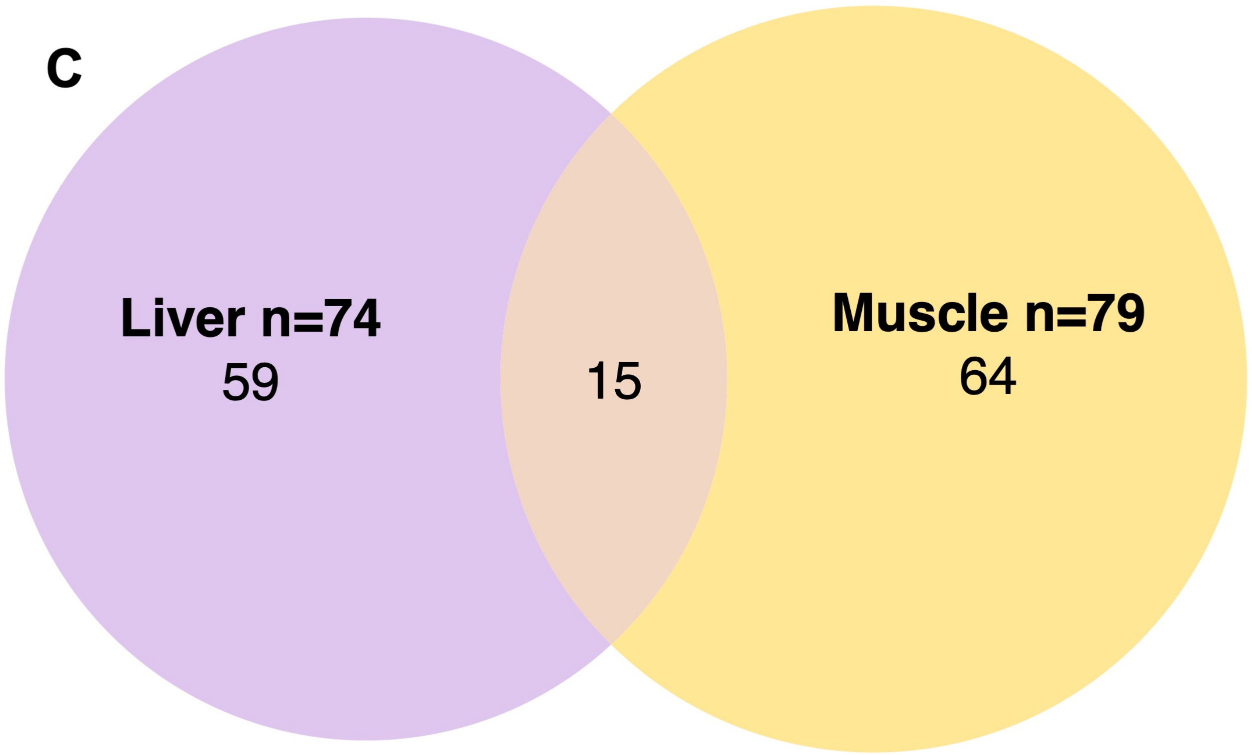

D

Common Pathways

NRF2-mediated Oxidative Stress Response
LPS/IL-1 Mediated Inhibition of RXR Function
Aryl Hydrocarbon Receptor Signaling
Xenobiotic Metabolism Signaling
Glutathione Redox Reactions I
Wound Healing Signaling Pathway
Xenobiotic Metabolism General Signaling Pathway
Oxytocin Signaling Pathway
Xenobiotic Metabolism CAR Signaling Pathway
Pulmonary Fibrosis Idiopathic Signaling Pathway
Uracil Degradation II (Reductive
Thymine Degradation
Xenobiotic Metabolism PXR Signaling Pathway
Hepatic Fibrosis / Hepatic Stellate Cell Activation
Xenobiotic Metabolism AHR Signaling Pathway
