# Supplementary material for: Restoring glucose balance: Conditional HMGB1 knockdown mitigates hyperglycemia in a Streptozotocin induced mouse model
Source: Heliyon. 2023 Dec 12;10(1):e23561. doi: 10.1016/j.heliyon.2023.e23561 (PMC10770459; doi:10.1016/j.heliyon.2023.e23561)
Supplement: Multimedia component 5 [file mmc5.pdf]

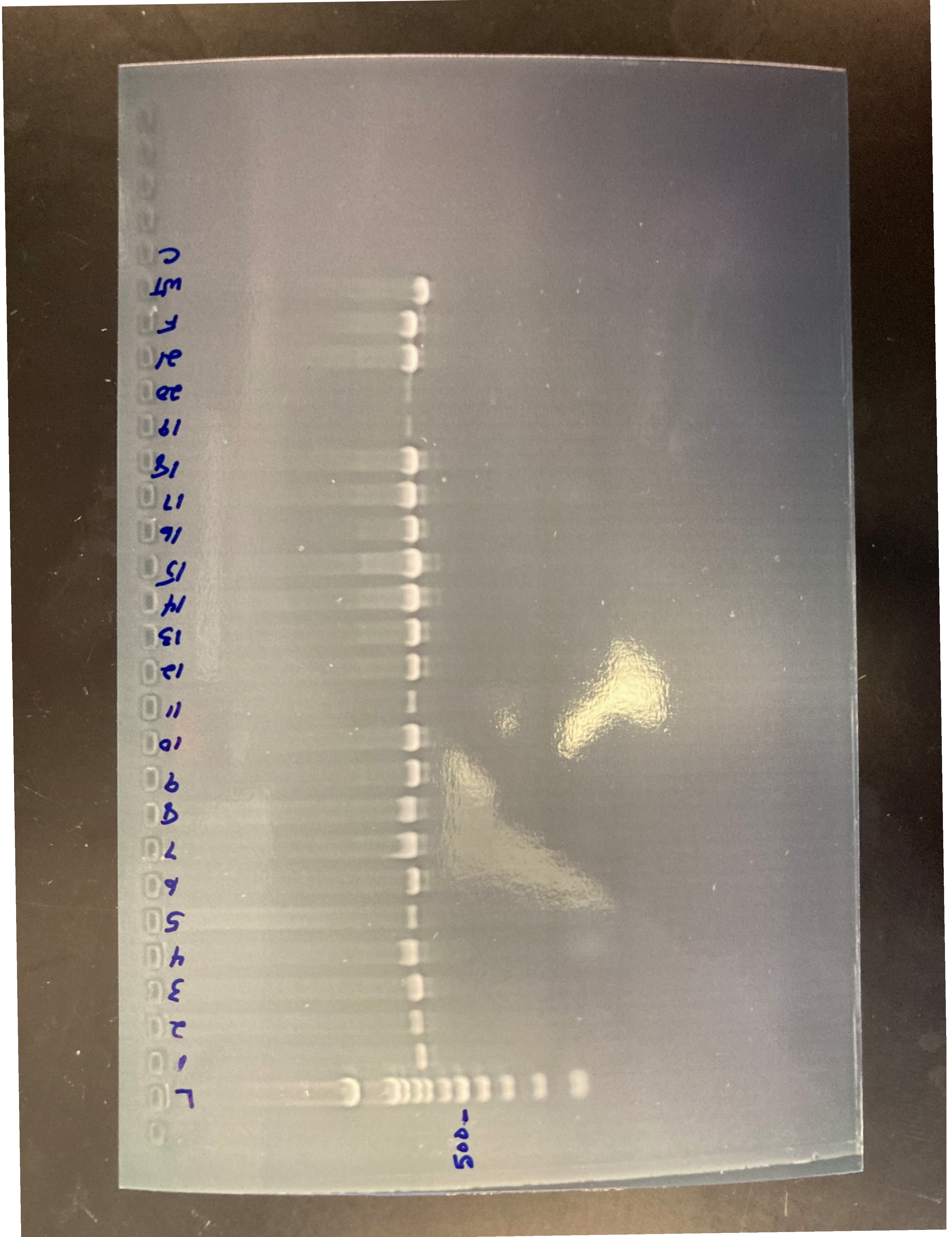

L
1
2
3
4
5
6
7
8
9
10
11
12
13
14
15
16
17
18
19
20
21
C
500-

IB: HMGB1 abcam 1:650
Tamoxifen Tx mice
4-12% NuPAGE Gel MES
NuPAGE™ LDS Sample Buffer (4X)
Nitrocellulose membrane
25 microliter per well

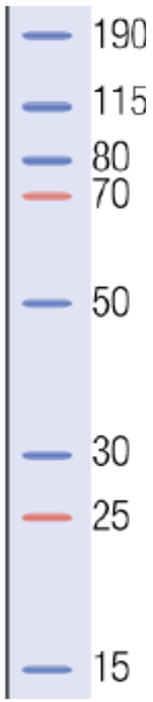

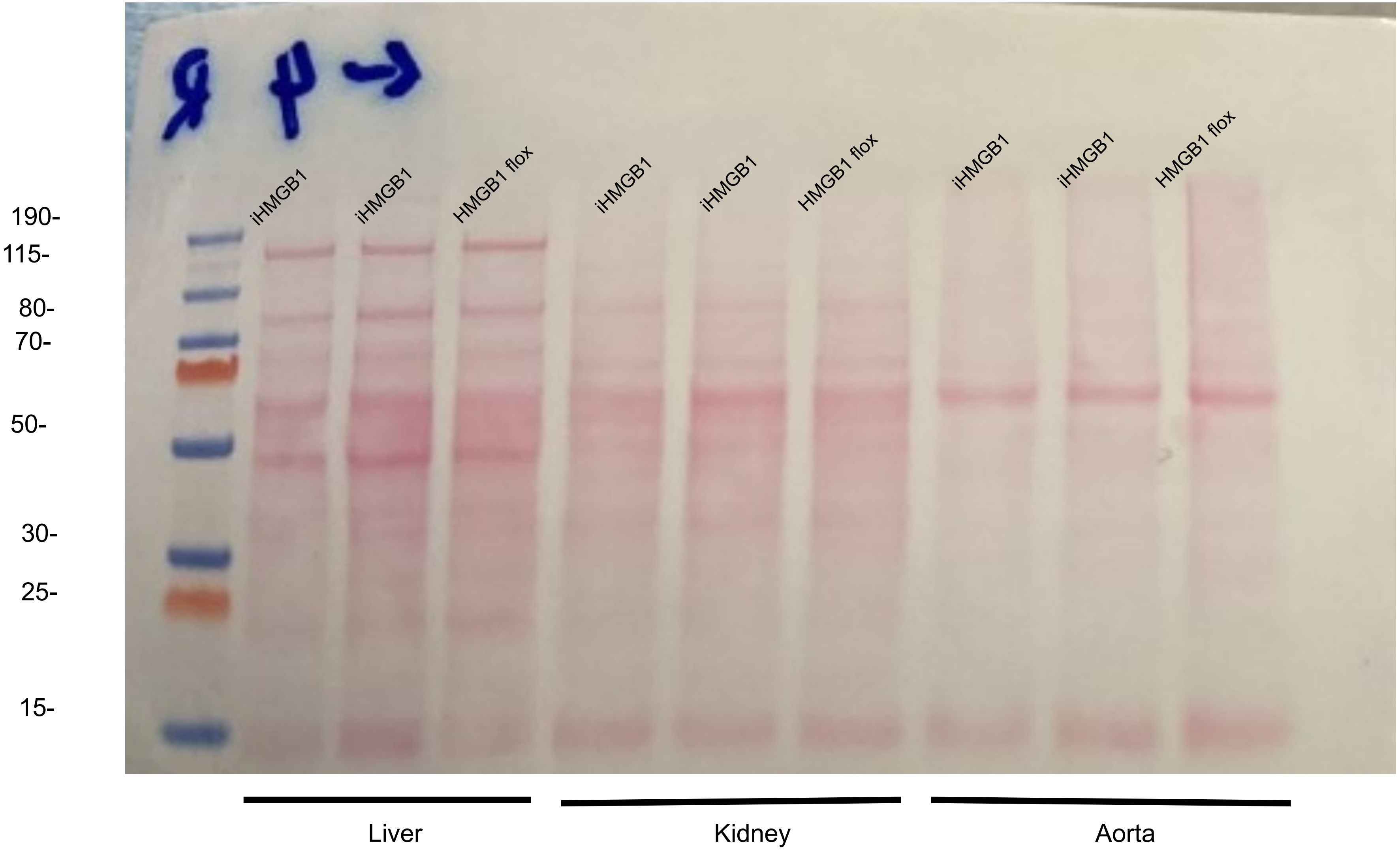

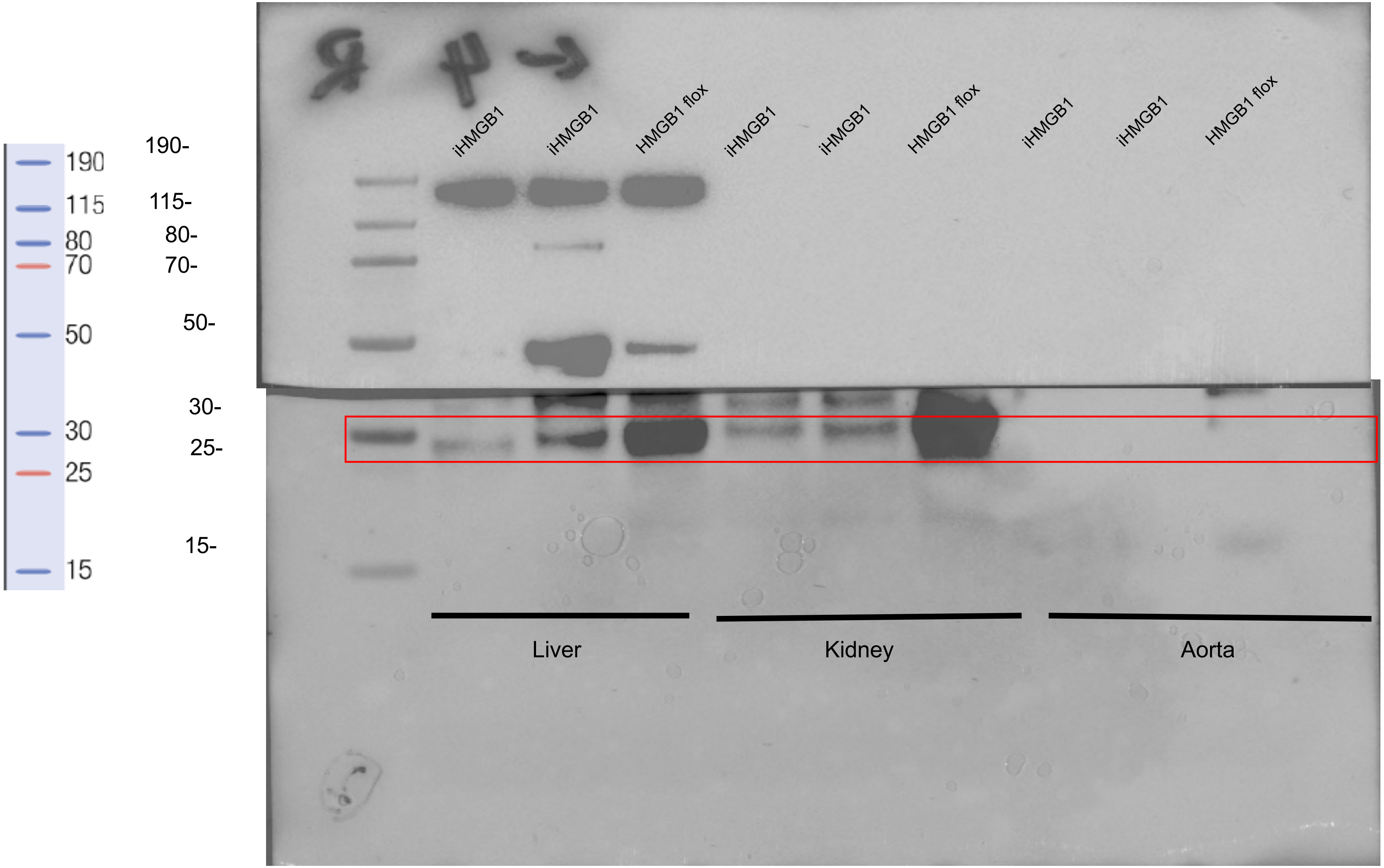

190
115
80
70
50
30
25
15
190-
115-
80-
70-
50-
30-
25-
15-
iHMGB1
iHMGB1
HMGB1 flox
iHMGB1
iHMGB1
HMGB1 flox
iHMGB1
iHMGB1
HMGB1 flox
Liver
Kidney
Aorta

IB: HMGB1 abcam 1:650
Tamoxifen Tx mice
4-12% NuPAGE Gel MES
NuPAGE™ LDS Sample Buffer (4X)
Nitrocellulose membrane
25 microliter per well

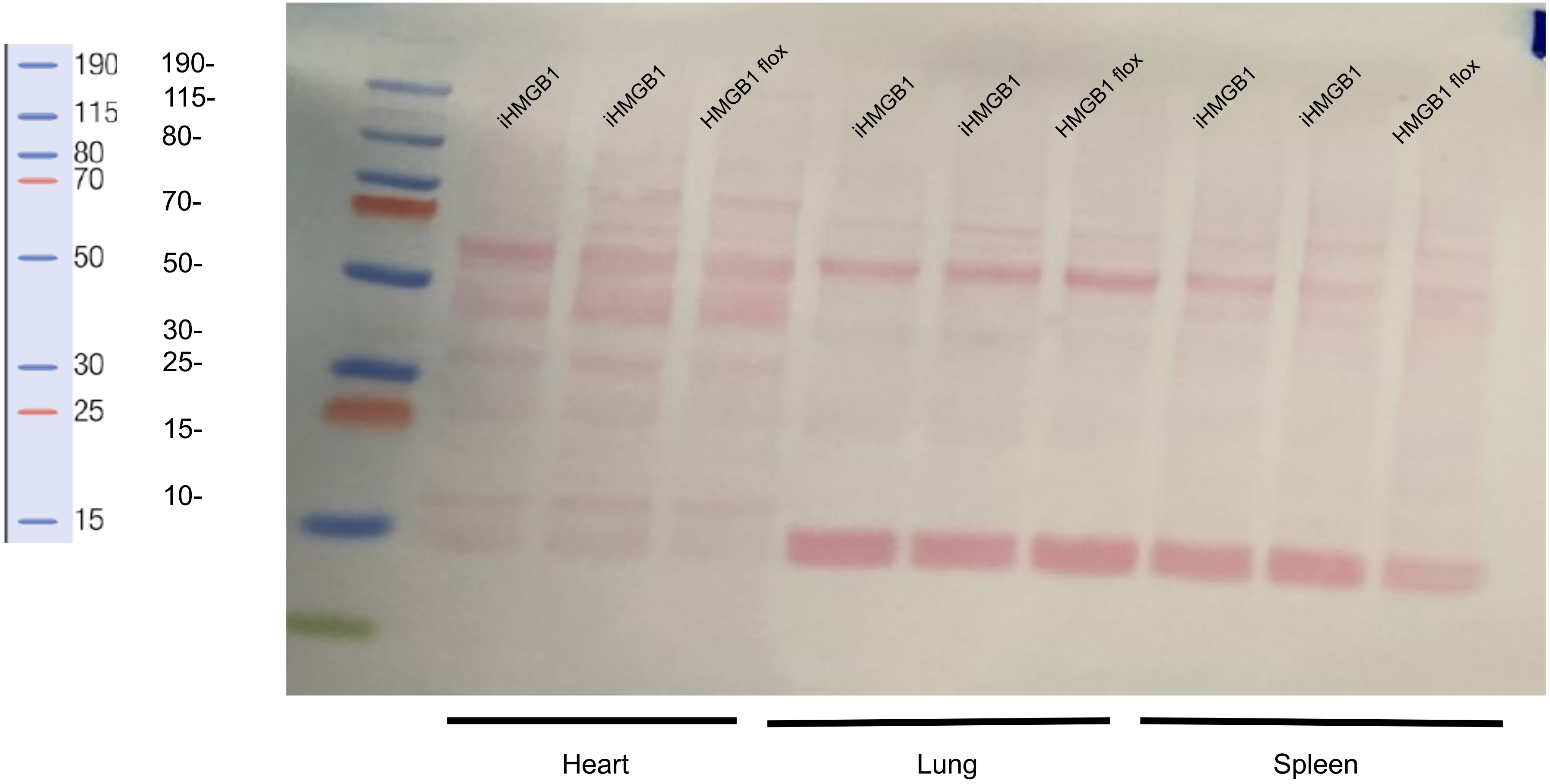

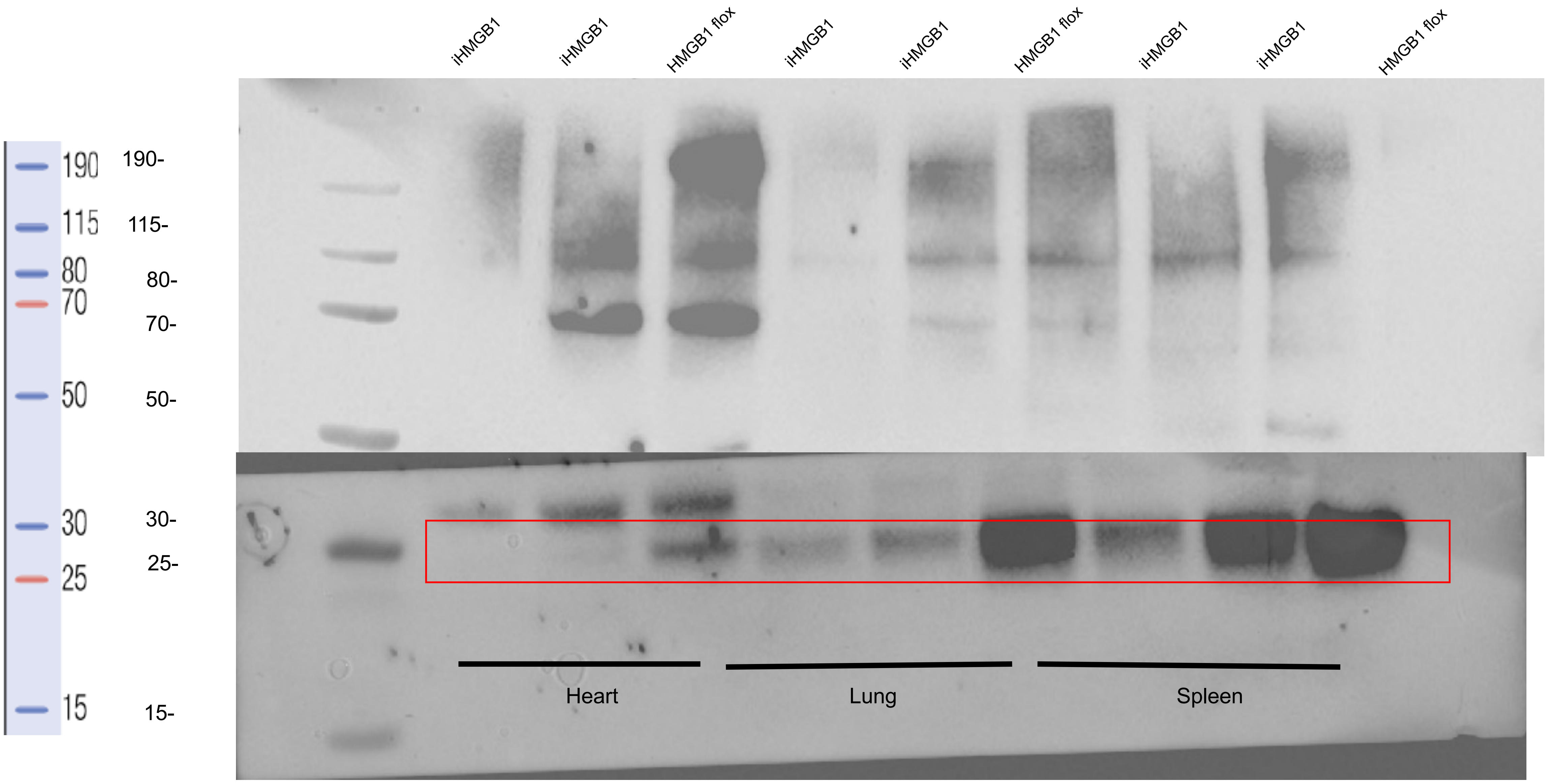

iHMGB1
iHMGB1
HMGB1 flox
iHMGB1
iHMGB1
HMGB1 flox
iHMGB1
iHMGB1
HMGB1 flox
190
115
80
70
50
30
25
15
190-
115-
80-
70-
50-
30-
25-
15-
Heart
Lung
Spleen
